# MOF-derived bimetallic nanozyme to catalyze ROS scavenging for protection of myocardial injury

**DOI:** 10.7150/thno.83543

**Published:** 2023-04-29

**Authors:** Kaiyan Xiang, Haoguang Wu, Yu Liu, Sheng Wang, Xueling Li, Bowei Yang, Yunming Zhang, Long Ma, Guangming Lu, Liangcan He, Qianqian Ni, Longjiang Zhang

**Affiliations:** 1 Department of Diagnostic Radiology, Nanjing Jinling Hospital, Affiliated Hospital of Medical School, Nanjing University, Nanjing, Jiangsu, China.; 2 Department of Diagnostic Radiology, Jinling Hospital, Nanjing Medical University, Nanjing, Jiangsu, China.; 3 Heart Center, Department of Cardiovascular Medicine, Zhejiang Provincial People's Hospital, Affiliated People's Hospital, Hangzhou Medical College, Hangzhou, Zhejiang, China.; 4 Department of Diagnostic Radiology, Yong Loo Lin School of Medicine, National University of Singapore, Singapore 117597, Singapore.; 5 Nanomedicine Translational Research Program, NUS Center for Nanomedicine, Yong Loo Lin School of Medicine, National University of Singapore, Singapore 117597, Singapore.; 6 School of Medicine and Health, Key Laboratory of Micro-systems and Micro-structures Manufacturing (Ministry of Education), Harbin Institute of Technology, Harbin, 150001 China.; 7 Clinical Imaging Research Centre, Centre for Translational Medicine, Yong Loo Lin School of Medicine, National University of Singapore, Singapore 117599, Singapore.

**Keywords:** reactive oxygen species, myocardial injury, nanozyme, metal-organic framework, nanomedicine

## Abstract

**Rationale:** Myocardial injury triggers intense oxidative stress, inflammatory response, and cytokine release, which are essential for myocardial repair and remodeling. Excess reactive oxygen species (ROS) scavenging and inflammation elimination have long been considered to reverse myocardial injuries. However, the efficacy of traditional treatments (antioxidant, anti-inflammatory drugs and natural enzymes) is still poor due to their intrinsic defects such as unfavorable pharmacokinetics and bioavailability, low biological stability, and potential side effects. Nanozyme represents a candidate to effectively modulate redox homeostasis for the treatment of ROS related inflammation diseases.

**Methods:** We develop an integrated bimetallic nanozyme derived from metal-organic framework (MOF) to eliminate ROS and alleviate inflammation. The bimetallic nanozyme (Cu-TCPP-Mn) is synthesized by embedding manganese and copper into the porphyrin followed by sonication, which could mimic the cascade activities of superoxide dismutase (SOD) and catalase (CAT) to transform oxygen radicals to hydrogen peroxide, followed by the catalysis of hydrogen peroxide into oxygen and water. Enzyme kinetic analysis and oxygen-production velocities analysis were performed to evaluate the enzymatic activities of Cu-TCPP-Mn. We also established myocardial infarction (MI) and myocardial ischemia-reperfusion (I/R) injury animal models to verify the ROS scavenging and anti-inflammation effect of Cu-TCPP-Mn.

**Results:** As demonstrated by kinetic analysis and oxygen-production velocities analysis, Cu-TCPP-Mn nanozyme possesses good performance in both SOD- and CAT-like activities to achieve synergistic ROS scavenging effect and provide protection for myocardial injury. In both MI and I/R injury animal models, this bimetallic nanozyme represents a promising and reliable technology to protect the heart tissue from oxidative stress and inflammation-induced injury, and enables the myocardial function to recover from otherwise severe damage.

**Conclusions:** This research provides a facile and applicable method to develop a bimetallic MOF nanozyme, which represents a promising alternative to the treatment of myocardial injuries.

## Introduction

Myocardial injury, one of the most common cardiovascular diseases (CADs), remains to be a major public health problem 1-4. The pathogenesis of myocardial injury induced by MI is usually connected with the overproduction of ROS and inflammation in the infarcted region and surrounding myocardium 5-8. Typically, mammalian cells have evolved antioxidant enzyme systems to neutralize the extra ROS and maintain cellular redox homeostasis, such as SOD, CAT, and glutathione peroxidases (GPx) 1,9. However, the activities of endogenous enzymatic antioxidants usually decrease over time, leading to the loss of redox equilibrium and massive accumulation of ROS 7,10,11. Excess ROS can overwhelm the cellular antioxidant capacity through lipid peroxidation, DNA damage, and protein oxidation, which finally induce necrotic cell death. Moreover, ROS can stimulate the expression of adhesion molecules (e.g., integrin β1, ICAM-1) and inflammatory cytokines (e.g., TNF-α, IL-1β), thus promoting the development of inflammation and immune response which in turn, will lead to the progression of heart failure 12,13. Thus, ROS scavenging and anti-inflammation are considered effective therapeutic strategies for MI treatment. However, traditional treatments including stoichiometric antioxidants and anti-inflammatory drugs are restricted due to unfavorable biocompatibility and pharmacokinetics 7,14. Although natural enzymes with high catalytic activity and substrate selectivity are also used for anti-inflammation, the intrinsic drawbacks of the high cost of manufacture and storage, biological instability, low recyclability, and potential immunogenicity hinder their clinical application 15-17. To tackle these challenges, the advancement of artificial catalysts has recently shown promising results to scavenge excess ROS within cardiac regenerative medicine 18-24.

Over the past few decades, nanomaterials with enzyme-mimicking characteristics (termed nanozymes) have emerged as excellent artificial catalysts to scavenge ROS. Nanozyme usually possesses nanoscale size (1-100 nm) and enzyme-like activities 25-27. The relatively low cost in production, facile synthesis process, tunable structures, and high biological stability of nanozymes make it a promising alternative for the treatment of ischemic heart diseases 15,28. Among multiple formations of nanozymes, metal-organic frameworks (MOFs) have drawn extensive attention in biomedical applications 29-31. MOFs are crystalline porous hybrids that consist of metal-based nodes and organic ligands that can mimic natural enzyme structures or functions 32-34. With brilliant characteristics of multiple catalytic centers, suitable sizes, intrinsic biocompatibility, and biodegradability, nanoscale MOFs have been widely applied in the treatment of cancers as well as ischemic diseases 35-37. Although coordination of metal ions/clusters has been fabricated to induce catalysis effects, the cascade reaction systems to mimic multiple enzymatic activities have mostly relied on multi-component strategies that be integrated into one nanoparticle 38-40, which will be hampered by complications in nanoparticle formation and large-scale production. Here, we designed and synthesized a single component, MOF-based nanozyme (denoted as Cu-TCPP-Mn) by introducing manganese and copper metal atoms into the tetrachloroporphyrin (TCPP), which shows integrated and high catalytic performance of SOD and CAT. Leveraging on the reversible one-electron oxidation between Mn (III) porphyrin and Mn (IV), this bimetallic nanozyme could thus catalyze ROS to hydrogen peroxide (H_2_O_2_), and then covert the produced H_2_O_2_ to O_2_. We successfully demonstrated that this bimetallic MOF nanozyme is a promising nanoplatform for ROS scavenging and inflammation inhibition, thus enabling long-term ventricular remodeling, facilitating heart function recovery, and protecting the myocardial tissues from ischemia damages in both myocardial infarction and myocardial and myocardial I/R injury animal models.

## Results and Discussion

### Synthesis and characterization of Cu-TCPP-Mn

**Figure 1:**
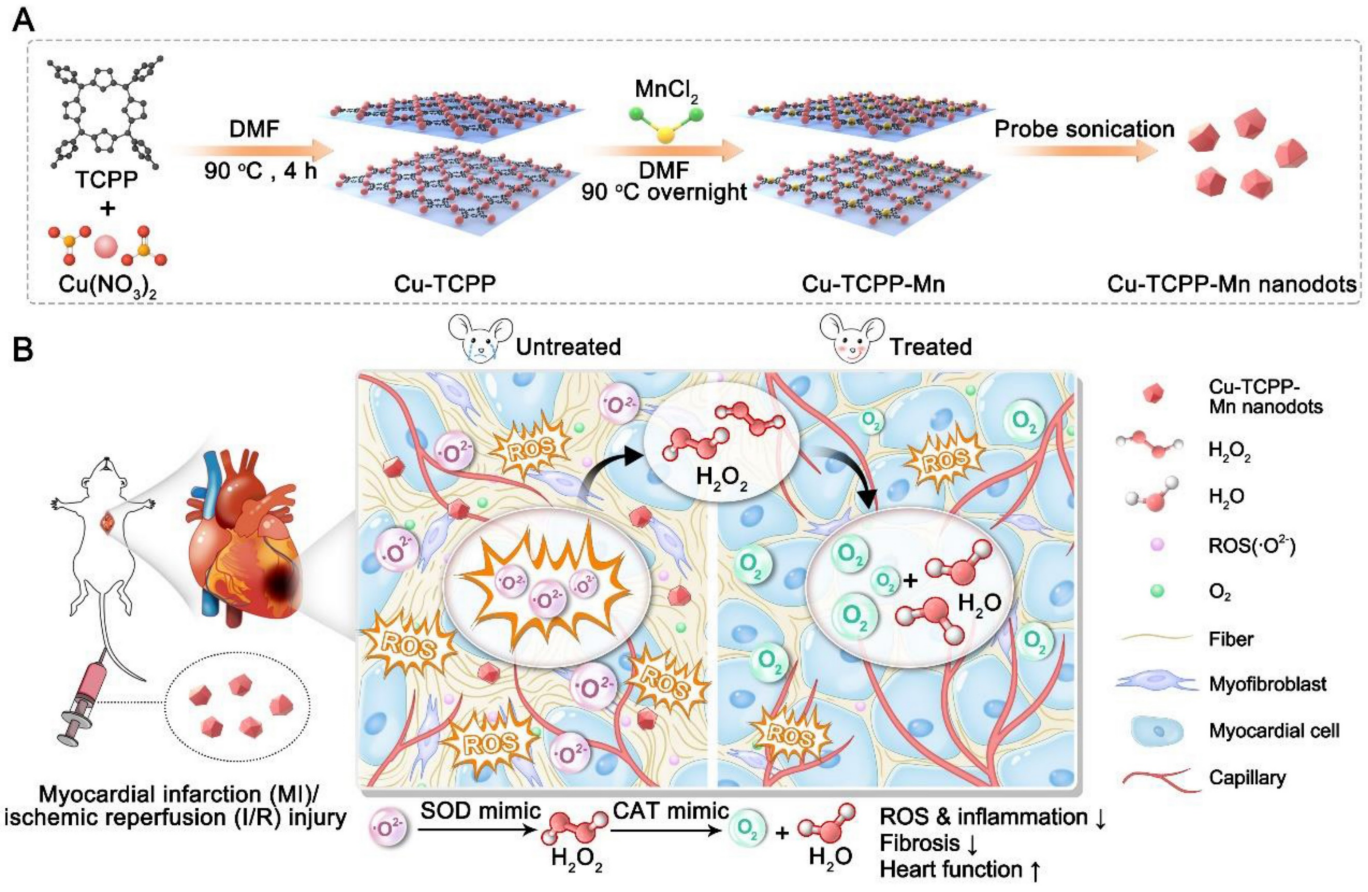
** Schematic illustration of the design and synthesis of Cu-TCPP-Mn nanozyme for myocardial injury treatment**. (A) The bimetallic Cu-TCPP-Mn nanozyme was fabricated by embedding manganese and copper into the porphyrin *via* solvothermal method, followed by sonication into small MOF nanodots. (B) Cu-TCPP-Mn nanozyme retained cascade activity that has been shown to scavenge ROS, inhibit inflammation, reduce myocardium fibrosis and promote constructive remodeling and vascularization in MI and I/R injury animal models.

**Figure 2:**
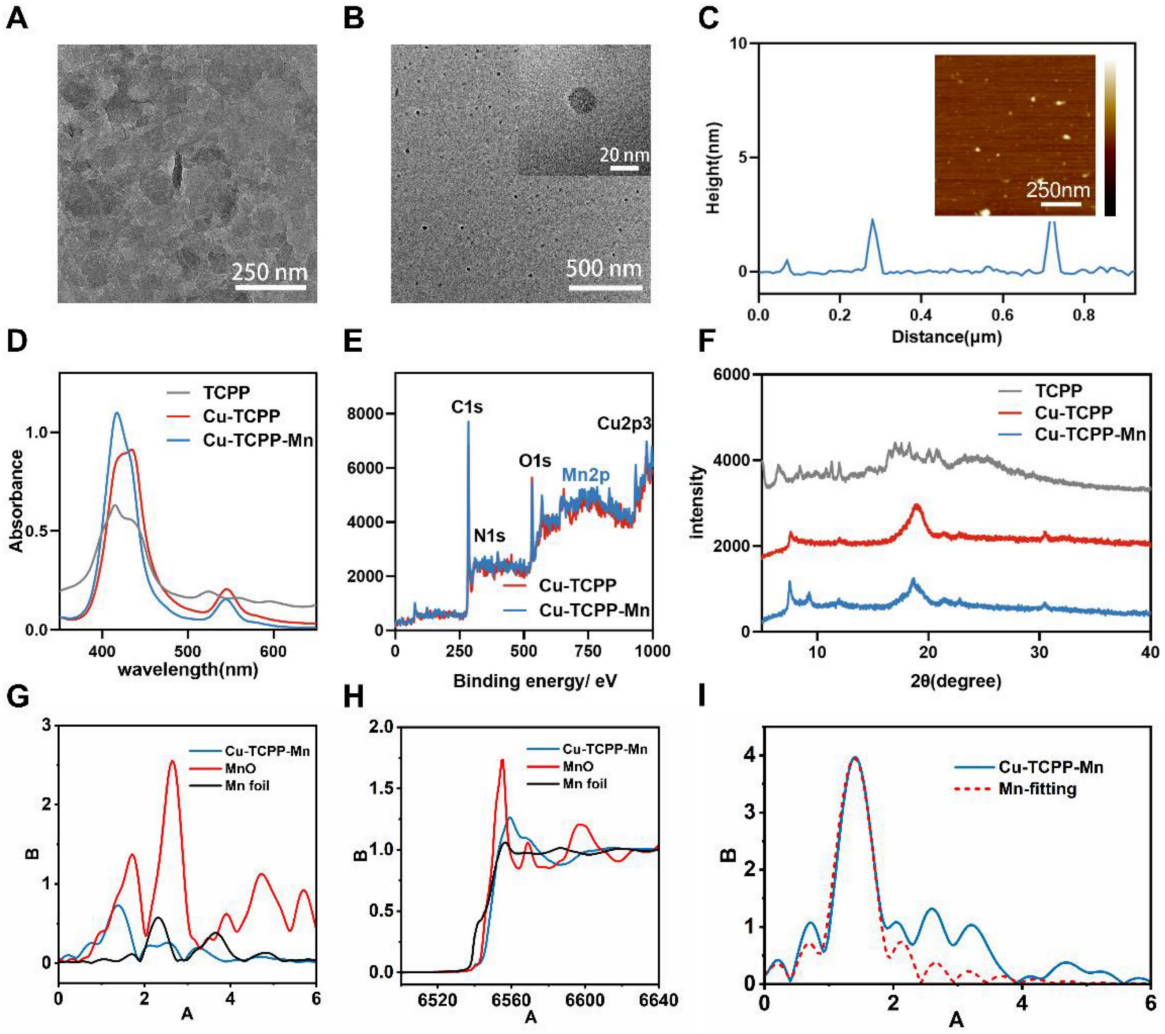
** Characterization of Cu-TCPP-Mn nanozyme.** (A) Representative TEM image of Cu-TCPP-Mn nanozyme with sheet-like morphology. (B) Representative TEM images of Cu-TCPP-Mn nanodots. (C) Quantitative analysis of the thickness of Cu-TCPP-Mn nanodots, and representative AFM image of Cu-TCPP-Mn nanodots (inserted). (D) UV-vis absorption spectra of TCPP, Cu-TCPP and Cu-TCPP-Mn. (E) XPS of Cu-TCPP and Cu-TCPP-Mn. (F) PXRD pattern of the TCPP, Cu-TCPP and Cu-TCPP-Mn. (G) Normalized Mn K-edge XANES spectra of different samples; (H) Fourier transform EXAFS spectra (k^3^-weighted) of Cu-TCPP-Mn; (I) Mn K-edge EXAFS of Cu-TCPP-Mn in the R space and the fitting curves without correcting for the scattering phase shift.

Cu-TCPP-Mn, the bimetallic MOF-based nanozyme to mimic SOD and CAT activities, was designed and synthesized as shown in Figure 1. Briefly, Cu-TCPP-Mn nanozyme was prepared in three steps: 1) Cu-TCPP MOF structures were synthesized by mixing TCPP and Cu(NO_3_)_2_ *via* the bottom-up method with the assistance of benzoic acid 39; 2) manganese ions (Mn^2+^) were doped into Cu-TCPP structures to formulate bimetallic Cu-TCPP-Mn nanosheets; 3) the resulting Cu-TCPP-Mn nanosheets were sonicated into small Cu-TCPP-Mn nanodots for *in vivo* myocardium injury therapy. The successful fabrication of Cu-TCPP was confirmed by transmission electron microscopy (TEM) and scanning electron microscopy (SEM) that displayed the ultrathin sheet-like morphology of the prepared Cu-TCPP structures (Figure S1A-B). Atomic force microscopy (AFM) revealed that the thickness of Cu-TCPP structure was 10.52 ± 0.21 nm (Figure S1C-D), which was consistent with previous reports 37,38,41. Afterwards, Mn^2+^ was introduced to the constructed Cu-TCPP by heating the mixture of MnCl_2_^.^4H_2_O and Cu-TCPP in N, N-dimethylformamide (DMF) solution at 90 °C overnight to formulate bimetallic MOF nanoparticles (Cu-TCPP-Mn). The as-synthesized Cu-TCPP-Mn maintained sheet-like morphology with particle size of ~100 nm (Figure 2A, Figure S2). After sonicating by ultrasound probe for 6 h, the Cu-TCPP-Mn nanodots were purified *via* centrifugation. TEM images exhibited highly dispersed nanodots with a uniform size of 20 nm (Figure 2B). Also, the as-synthesized Cu-TCPP-Mn presented good stability in buffers with different pH values (Figure S3). AFM indicated that the average thickness of Cu-TCPP-Mn nanodots was 2.34 ± 0.06 nm (Figure 2C). UV-Vis spectroscopy showed that Cu-TCPP-Mn nanodots exhibited the absorption peaks at 420 and 540 nm, while Cu-TCPP displayed absorption peak at 434 nm (Figure 2D) 42. The chemical composition of Cu-TCPP-Mn nanozyme was further confirmed by X-ray photoelectron spectroscopy (XPS), showing the existence of Mn at the peak of 397.725 eV and reduction of pyrrolic nitrogen at 400.900 eV of Cu-TCPP and Cu-TCPP-Mn (Figure 2E, Figure S4) 38. The powder X-ray diffraction (PXRD) showed that Cu-TCPP-Mn had a characteristic peak and higher crystallinity (Figure 2F). According to inductively coupled plasma-atomic emission spectrometry (ICP-AES), the content of Mn and Cu in the Cu-TCPP-Mn nanozyme were 2.89% and 11.83%, which was similar to the previous report (Table S1-S2) 43-45, while Cu in Cu-TCPP was 14.13%, implying the fact that addition of Mn^2+^ could replace the chelation of Cu^2+^. We speculate that the Mn^2+^ could coordinate with four N atoms, which represented the central porphyrin linker 43,46,47. To verify it, X-ray absorption near edge structure (XANES) spectroscopy and extended X-ray absorption fine structure (EXAFS) spectroscopy were performed and demonstrated that Cu-TCPP-Mn nanozyme had a well-defined MnN_4_ chemical structure (Figure 2G-I, Table S3). The coordination peak of Cu-O both in Cu-TCPP and Cu-TCPP-Mn was also confirmed by Cu K-edge EXAFS and XANES (Figure S5). Zeta potential of Cu-TCPP-Mn were -14.8 ± 2.12 mV (Figure S6). Fourier transform infrared (FTIR) spectrometer further confirmed the chemical compositions of the nanozyme (Figure S7). Cu-TCPP showed several peaks between 1720 and 1200 cm^-1^, representing the symmetric vibration mode of C=O (1606 cm^-1^), asymmetric vibration mode of O=C-O (1661, 1403 cm^-1^) and symmetric vibration mode of O=C-O (1342 cm^-1^) 37,48. These characterizations indicated the coordination of the carboxyl group in the TCPP ligand to the Cu atom, which led to the asymmetric stretch of the benzene ring 38. A left shift peak at 999 cm^-1^ and vanished peak around 1714 cm^-1^ of Cu-TCPP-Mn nanozyme confirmed the incorporation of Mn(II) with TCPP ligand. These results indicated the successful synthesis of Cu-TCPP-Mn nanozyme.

### *In vitro* cascade enzyme mimicking activities of Cu-TCPP-Mn nanozyme

**Figure 3:**
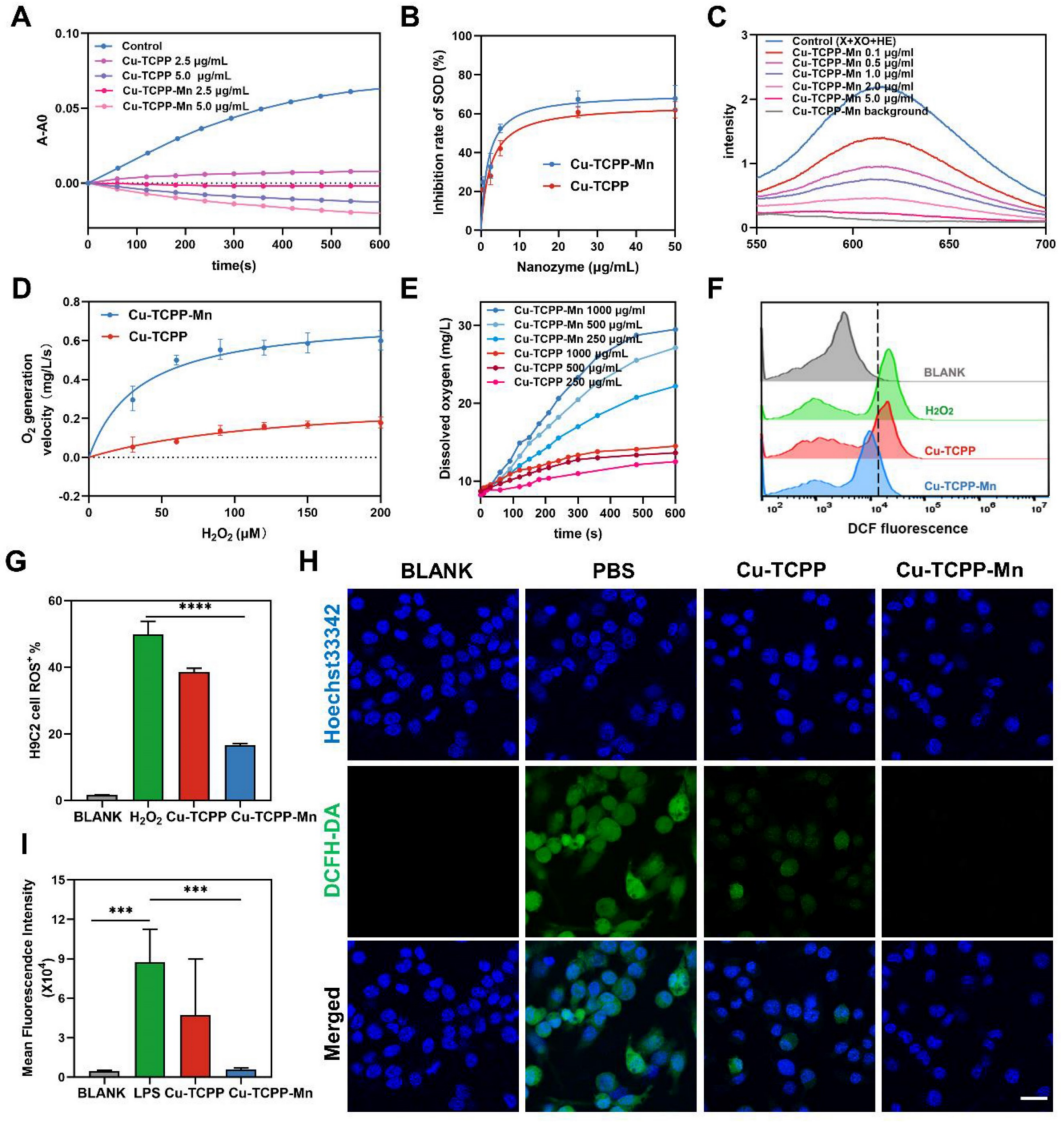
***In vitro* ROS-scavenging activities of Cu-TCPP-Mn nanozyme.** (A) Kinetic curves of A-A_0_ (550 nm) with X and XO treated with different concentrations of Cu-TCPP-Mn by monitoring the reduction of NBT. (B) Inhibition rate of SOD of Cu-TCPP and Cu-TCPP-Mn calculated by NBT kinetic assay. (C) Fluorescent spectra of the mixture of DHE, X, and XO treated with different concentrations of Cu-TCPP-Mn. (D) Kinetics of O_2_ generation velocity for CAT-like activity of Cu-TCPP and Cu-TCPP-Mn nanozyme. (E) Typical kinetic curves of dissolved oxygen generated from the decomposition of H_2_O_2_ after treatment with different concentrations of Cu-TCPP-Mn (250, 500, 1000 μg). (F) ROS evaluation in H_2_O_2_ (100 μM) pre-treated H9C2 cells by flow cytometry with the treatment of Cu-TCPP and Cu- TCPP-Mn (5 μg/mL). (G) Quantitative analysis of ROS production in H9C2 cells treated with Cu-TCPP and Cu-TCPP-Mn (5 μg/mL). (H) Representative fluorescence images of ROS staining (DCFH-DA, green fluorescence) of RAW 264.7 cells treated with Cu-TCPP and Cu-TCPP-Mn and (I) quantitative analysis of mean fluorescence intensity. Scale bar, 20 μm. Data are presented as mean ± standard deviation (S.D.) (n = 5). The comparisons between samples were operated by one-way ANOVA. *** indicates P < 0.001; **** indicates P < 0.0001.

After fully characterizing Cu-TCPP-Mn nanodots, we evaluated their capacity to mimic cascade SOD and CAT activity *in vitro*. Since it is widely accepted that scavenging ^.^O_2_^-^ is an essential step in the anti-ROS cascade reaction system, we first investigated their SOD-like activity by monitoring the elimination of ^.^O_2_^-^ 49. Nitrotetrazolium blue chloride (NBT), a redox indicator for the detection of ^.^O_2_^-^, was applied to evaluate the SOD activity of the developed bimetallic Cu-TCPP-Mn nanozymes. By mixing xanthine (X) and xanthine oxidase (XO) with or without nanozymes, the generation and elimination of ^.^O_2_^-^ can be dynamically detected by NBT at the absorbance of 550 nm 15. As shown in Figure 3A-B, Cu-TCPP and Cu-TCPP-Mn nanozymes both exhibited SOD-like activities and were in concentration-dependent manners. Furthermore, the SOD-like activities of nanozymes at different pH and temperature conditions were investigated (Figure S8). Interestingly, Cu-TCPP and Cu-TCPP-Mn remained steady SOD-mimicking activities under the environmental range of pH 5.5-8.8 and 20-37 °C (Figure S8A-F). Also note that Cu-TCPP-Mn nanodots exhibited slightly higher SOD-like activity than Cu-TCPP by comparison of inhibition rate under a wide range of conditions, which was likely due to the obtained Mn-N active sites that endowed Cu-TCPP-Mn with higher SOD-like activities (Figure S8G-J) 31,50,51. Additionally, the SOD-mimicking activities of Cu-TCPP-Mn were evaluated by another redox indicator, dihydroethidium (DHE), which has been used as a superoxide probe for ROS detection. By measuring the fluorescence signal at 610 nm of DHE, Cu-TCPP-Mn nanozyme alone showed subtle superoxide production, while Cu-TCPP-Mn was found to scavenge oxygen radicals produced by xanthine (X) and xanthine oxidase (XO) effectively, verifying that Cu-TCPP-Mn nanozymes possess excellent SOD-like activities (Figure 3B, Figure S9), as the coordination environment of MnN_4_ structure in Cu-TCPP-Mn is analogous to those natural Mn-SOD and catalase with functional metal centers 28,49. Moreover, compared with Cu-TCPP-Mn nanosheet structures, the nanodots presented superior dispersity and more exposed active sites owing to their biomimetic size 37, making them more like natural enzymes.

As the downstream product after disproportioning ^.^O_2_^-^, H_2_O_2_ was further catalyzed by CAT into H_2_O and O_2_. We then tested the consumption of H_2_O_2_ and the generation of O_2_ to validate the CAT-mimicking activities of Cu-TCPP-Mn nanozyme. It was revealed that Cu-TCPP-Mn could significantly eliminate H_2_O_2_ as detected by [Ru(dpp)_3_]^2+^Cl_2,_ which had better catalytic activity than Cu-TCPP (Figure S10). Additionally, the elimination rates of Cu-TCPP-Mn were found to be concentration dependent, where a larger amount of Cu-TCPP-Mn could significantly accelerate the catalysis of H_2_O_2_. Furthermore, the CAT-like kinetics calculated by the generation of dissolved oxygen verified that Cu-TCPP-Mn nanozyme enabled excellent CAT-like activity to catalyze H_2_O_2_ into water and O_2_ (Figure 3D) 35. The K_m_ value of Cu-TCPP-Mn with H_2_O_2_ as substrate was relatively four times lower than Cu-TCPP, V_max_ and K_cat_ of Cu-TCPP-Mn were significantly higher than that of Cu-TCPP, suggesting the higher CAT-like activity of Cu-TCPP-Mn (Table S4). In addition, the apparent affinity of Cu-TCPP-Mn (34.65 mM) is comparable to natural catalase with hydrogen peroxide as substrate (25 mM), demonstrating that MOF based nanozyme is an ideal catalyst candidate for biomedical applications 52-54. It is also found that higher concentration of Cu-TCPP-Mn could lead to rapid and more distinct oxygen production (Figure 3E). When incubated in buffers with different pH values, Cu-TCPP-Mn maintained relatively steady velocity to catalyze H_2_O_2_, highlighting its superior catalytic capability (Figure S11). Collectively, the bimetallic nanozyme of Cu-TCPP-Mn showed stable SOD- and CAT-like activities under different conditions.

### Synergistic ROS-scavenging effect at the cellular level

To investigate the antioxidant enzyme-like effect of Cu-TCPP-Mn nanozyme *in vitro*. 100 μM H_2_O_2_ was added to H9C2 cells for intracellular stimulation of ROS to mimic oxidative stress induced by myocardial ischemia. As shown in Figure 3F-G, the intracellular ROS level of Cu-TCPP-Mn treated group was remarkedly decreased as tested by flow cytometry. Moreover, LPS stimulated RAW 264.7 cells were used to test the anti-inflammation effect of Cu-TCPP-Mn. Laser scanning confocal fluorescence microscope (LSCFM) showed green fluorescence signals in the LPS treated (PBS) group, which represented elevated intracellular ROS stained by the H_2_DCFH-DA probe. Notably, the fluorescence signals were significantly decreased after the treatment of Cu-TCPP-Mn as compared with the LPS treated group, verifying the effective ROS scavenging capability of Cu-TCPP-Mn nanozyme (Figure 3H-I). Moreover, the intracellular ROS scavenging of Cu-TCPP-Mn nanozyme was further validated by spectrofluorimetry as shown in Figure S12. Consistent with the findings on confocal fluorescence images, ROS level of the Cu-TCPP-Mn group was decreased compared with the PBS group. Cu-TCPP-Mn nanozyme presented immunostimulatory effects as shown by the increased expression of immunostimulatory factors in macrophages (Figure S13). In addition, we investigated the anti-inflammation capabilities of Cu-TCPP-Mn. The ELISA results showed that less IL-1β and TNF-α production, and relatively higher IL-10 were detected after the treatment of Cu-TCPP-Mn, verifying Cu-TCPP-Mn could significantly inhibit the inflammation response (Figure S14).

Cytoprotective properties against ROS damage by Cu-TCPP-Mn nanozyme were tested in H9C2 (rat cardiomyocytes) and bEnd.3 (mouse cerebrovascular endothelial) cell lines by co-incubating with H_2_O_2_ to induce ROS production *in vitro*. As shown in Figure S15, the Cu-TCPP-Mn treatment significantly improved cell survival as tested by methyl thiazolyl tetrazolium (MTT) assay. Consistently, the flow cytometry results of propidium iodide (PI) apoptosis staining exhibited the remarkable cytoprotective effect of Cu-TCPP-Mn nanozyme, as determined by the decreased percentage of apoptotic cells (Figure S16). Finally, the cytotoxicity of Cu-TCPP-Mn was studied by MTT assay, showing that both Cu-TCPP and Cu-TCPP-Mn exhibited no evident cytotoxicity with concentrations under 50 μg/mL in RAW264.7 and bEnd.3 cells (Figure S17). The hemolysis ratio of Cu-TCPP-Mn was lower than 1% with different concentrations ranging from 1 μg/mL to 1000 μg/mL, indicating the benefits of Cu-TCPP-Mn nanozyme for biomedical applications (Figure S18). We further investigated the subcellular localization of the nanozyme. Based on previous studies, endolysosomes are major organelles for cellular internalization of nanozymes 55-57. Besides, mitochondria are primary organelles to produce ROS under oxidative stress 17,58,59. Herein, we hypothesize that Cu-TCPP-Mn nanozymes with ultrasmall particle size were released from endolysosome and then internalized by mitochondria to scavenge the generated ROS. H9C2 cells were co-incubated with Cy5-labeled Cu-TCPP-Mn, with cellular mitochondria stained with MitoTracker and lysosome stained with LysoTracker. LSCFM images of cellular uptake showed that the accumulation of Cu-TCPP-Mn was obviously observed in cytoplasm, with the Pearson's correlation coefficient of 0.521 in mitochondria and 0.317 in lysosome (Figure S19).

### *In vivo* anti-inflammation effect of Cu-TCPP-Mn nanozyme on MI mouse models

**Figure 4:**
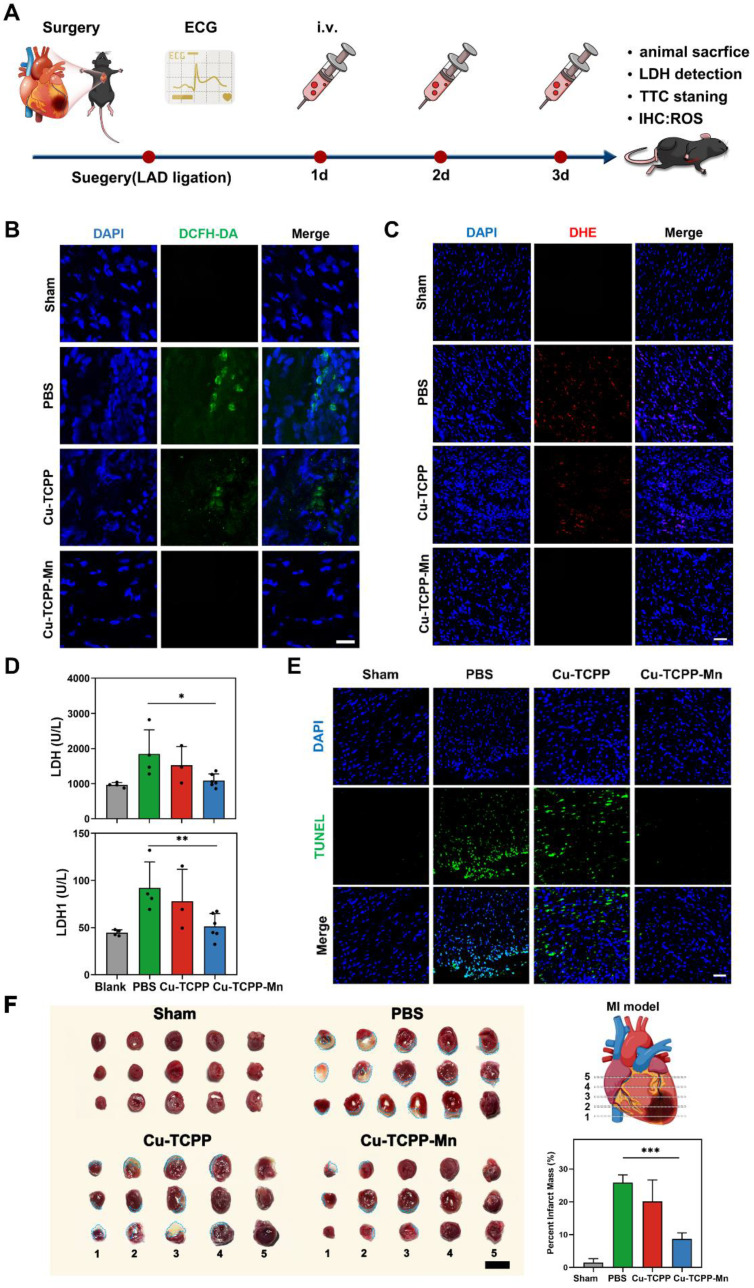
***In vivo* anti-inflammation effect of Cu-TCPP-Mn nanozyme on MI mice.** (A) The schematic of the study design. Representative ROS staining images of heart tissues harvested from MI mice models treated with PBS, Cu-TCPP and Cu-TCPP-Mn using DCFH-DA probe (B, Scale bar, 25 μm) and DHE probe (C, Scale bar, 50 μm). (D) the LDH and LDH1 concentrations of mouse serum with different treatment. (E) Immunofluorescent staining of TUNEL in the ischemic heart after different treatments. Scale bar, 50 μm. (F) Representative TTC staining images and quantitative data of Sham or MI heart slices treated with PBS, Cu-TCPP and Cu-TCPP-Mn, with the infarct area shown in blue dashed line. Scale bar, 100 mm. Data are presented as mean ± S.D. (n = 5). The comparisons between samples were operated by one-way ANOVA. * indicates P < 0.05; ** indicates P < 0.01; *** indicates P < 0.001; ns indicates P > 0.05 with no significance.

Acute MI is often associated with an intense inflammatory response at the early stage of pathogenesis. Ischemia-mediated ROS generation plays an important role in the activation of inflammatory signals in ischemic myocardium to induce cytokine and chemokine expression and promote leukocyte integrin activation 60,61. Inspired by the *in vitro* results of Cu-TCPP-Mn nanozymes in ROS scavenging, we thereby evaluated the ROS scavenging and anti-inflammatory efficacy of Cu-TCPP-Mn nanozyme on acute MI mouse models. The overall schematic of the study design was shown in Figure 4A. The mouse model of acute MI was established by permanent ligation of the left anterior descending (LAD) coronary artery 62-64. Successful LAD ligation was confirmed by an electrocardiogram (ECG) with the presence of ST-segment elevation (Figure S20, red arrow). To evaluate the biodistribution and heart accumulation of Cu-TCPP-Mn, Cy5 labeled Cu-TCPP-Mn nanozyme was injected into mice *via* the tail vein. At 6 h and 12 h post-injection, the heart and major organs from MI or sham groups were harvested for *ex vivo* fluorescent imaging. As shown in Figure S21A, prominent retention of Cu-TCPP-Mn was observed in liver after 6 and 12 h post administration. In addition, the fluorescence signal of heart in MI group can be distinctly observed after 6 and 12 h post injection. Specifically, more Cu-TCPP-Mn nanoparticles were accumulated in the heart of MI mice as compared with that of sham group, which was largely due to the enhanced permeability and retention (EPR) effect in inflammatory lesions of ischemic heart (Figure S21B-C). Next, the accumulation of Cu-TCPP-Mn nanozyme in major organs was also detected by ICP-AES. As shown in Figure S22, the liver exhibited the highest normalized dosage distribution of Cu-TCPP-Mn in both sham and MI mice groups (16.315 ± 2.147%ID/g versus 16.312 ± 1.021%ID/g). Specifically, the accumulation of Cu-TCPP-Mn in the MI heart was 8.062 ± 1.378%ID/g, which was significantly higher than that of sham heart (4.435 ± 0.453%ID/g). Taken together, nanozymes possessed preferential targeting to the inflammatory lesions of injured hearts 65-67. Then, MI mouse models were divided randomly into 4 groups (n = 5), including a healthy control group (Sham), MI surgery group treated with PBS (MI + PBS, 200 μL), Cu-TCPP nanozyme (MI + Cu-TCPP, 0.5 mg/kg) and Cu-TCPP-Mn nanozymes (MI + Cu-TCPP-Mn, 0.5 mg/kg). PBS and nanozymes were administered intravenously immediately after MI surgery (day 0), followed by the second injection at day 2 and third injection at day 3. After the last injection, the production of ROS in infarcted myocardium was detected by DHE and DCFH-DA, respectively. The treatment of Cu-TCPP-Mn profoundly decreased the production of ROS in ischemic myocardium (Figure 4B-C). Meanwhile, the concentration of lactate dehydrogenase (LDH) and lactate dehydrogenase-1 (LDH-1) in the serum, which are the major biomarkers that reflect ischemic severity, were also decreased in the Cu-TCPP-Mn-treated group (Figure 4D). Moreover, terminal deoxynucleotidyl transferase dUTP nick-end labeling (TUNEL) staining of infarcted myocardium after 3 days was also performed to evaluate the apoptosis of cardiomyocytes. As shown in Figure 4E, MI surgery caused increased apoptosis of cardiomyocytes compared with the sham group. 3 days after the treatment of Cu-TCPP-Mn nanozyme, the number of TUNEL-positive cardiomyocytes was significantly decreased, which was consistent with intracellular findings that Cu-TCPP-Mn possessed protective effect in H9C2 cardiomyocytes and RAW264.7 cells *via* ROS scavenging. Tetrazolium chloride (TTC) staining also showed that the infarct size (white-stained areas, blue dashed line) was reduced conspicuously (p < 0.05) after the administration of Cu-TCPP-Mn compared with PBS or Cu-TCPP groups (Figure 4F). These results demonstrated that cardiomyocyte death was ameliorated by Cu-TCPP-Mn nanozyme.

### The long-term impact of Cu-TCPP-Mn on cardiac remodeling in MI mouse models

**Figure 5:**
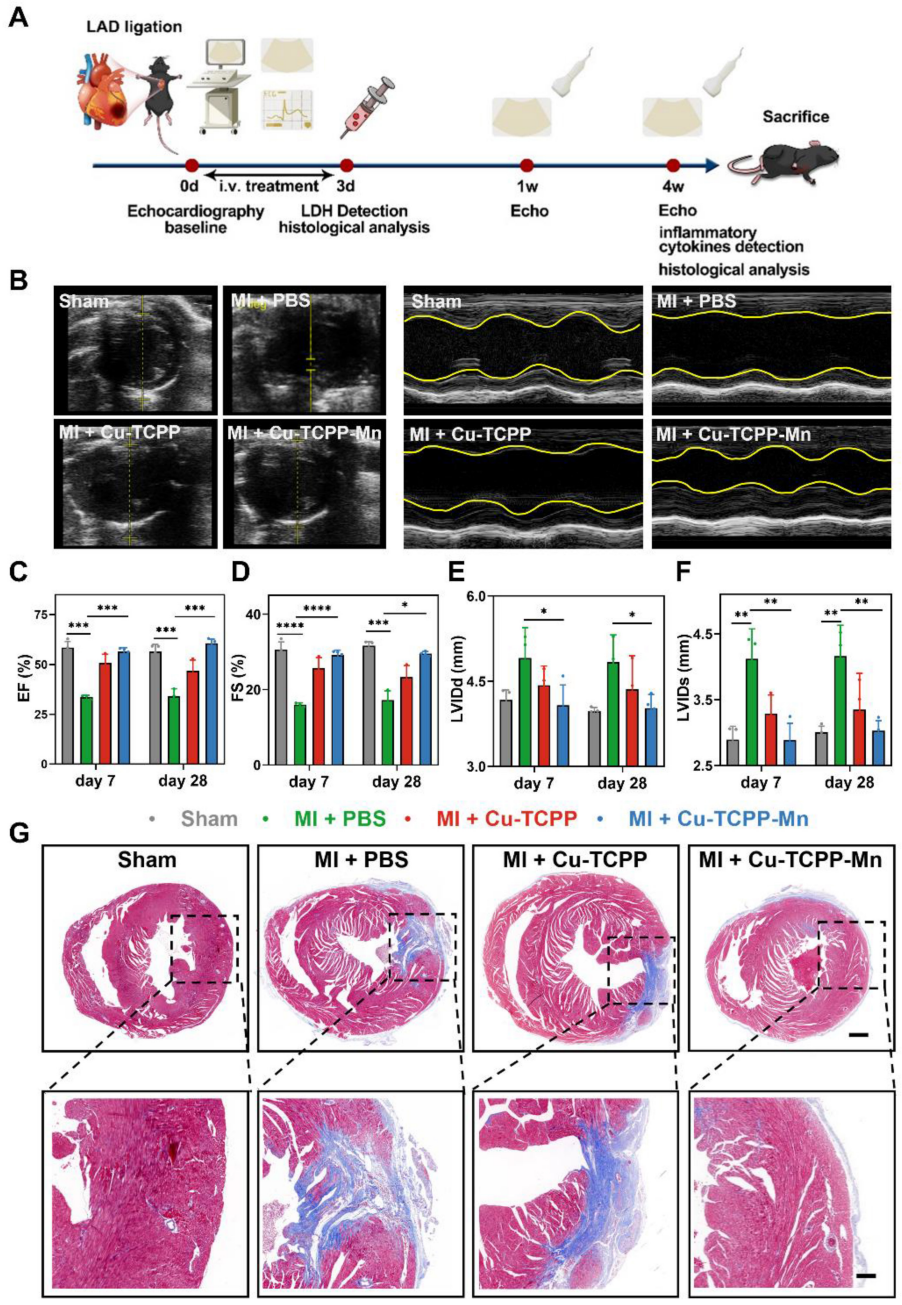
** Therapeutic efficacy of Cu-TCPP-Mn on MI mice.** (A) The schematic of the study design. (B) Representative echocardiographic images and corresponding M-mode images of MI mouse models at day 7 post treatments. The yellow wavy line (right panel) in the top and bottom area indicates left ventricular wall thickness and left ventricular posterior wall thickness. Distance between two wavy lines indicates left ventricular dimension. (C) Ejection fraction (EF) and (D) fractional shortening (FS) were evaluated by echocardiography after different treatments at one week and four weeks, respectively (n = 3-4 per group). (E) Left ventricular end diastolic dimension (LVIDd) and (F) left ventricular end systolic dimension (LVIDs) were evaluated by echocardiography after different treatments at one week and four weeks, respectively (n = 3-4 per group). (G) Representative Masson's trichrome staining images of infarcted hearts 4 weeks after injection (blue represents scar tissue; red represents viable myocardium). Scale bar, 500 μm (top), 150 μm (bottom). Data are presented as mean ± S.D. The comparisons between samples were operated by one-way ANOVA. * indicates P < 0.05; ** indicates P < 0.01; *** indicates P < 0.001; ns indicates P > 0.05 with no significance.

After acute MI-induced pathological damage, the loss of cardiomyocytes and gradual formation of scar would impair the cardiac function and even lead to heart failure 68. To gain further insight into the long-term protective effects of nanozyme on heart function and cardiac remodeling in MI mice, we used echocardiography to monitor the cardiac function after MI surgery at indicated time points (Figure 5A). Figure 5B exhibited that, at day 7 post-surgery, MI surgery caused thinning of the cardiac anterior/posterior walls, accompanied by systolic and diastolic cardiac dysfunctions. With the treatment of Cu-TCPP-Mn, the left ventricular ejection fraction (LVEF), LV fractional shortening (LVFS), diastolic left ventricular internal diameter (LVIDd), as well as systolic left ventricular internal diameter (LVIDs) were significantly improved after a week, and the effectiveness of restoration was maintained 4 weeks after the treatments (Figure 5C-F). Notably, the treatment of Cu-TCPP could transitorily restore the EF and FS at day 7. However, the cardiac function of Cu-TCPP group (measured by EF and FS) at day 28 got worse as compared with Cu-TCPP-Mn, indicating the superiority of Cu-TCPP-Mn for the treatment of MI. These results verified that Cu-TCPP-Mn nanozyme outperformed Cu-TCPP with reliable protective effects not only in the acute phase, but also in the long-term recovery of cardiac function. Next, histological analysis by Masson's trichrome staining was carried out to investigate the cardiac remodeling after MI. As shown in Figure 5G, the distribution of scar tissue (blue) in the dissected heart could be easily observed in the PBS group, and the size of scar tissues was evidently reduced in Cu-TCPP and Cu-TCPP-Mn treated group. Cu-TCPP-Mn group showed less fibrosis and better cardiac morphology compared to all other groups. The above results demonstrated that the treatment of Cu-TCPP-Mn nanozyme can improve heart function and promote heart repair after MI.

**Figure 6:**
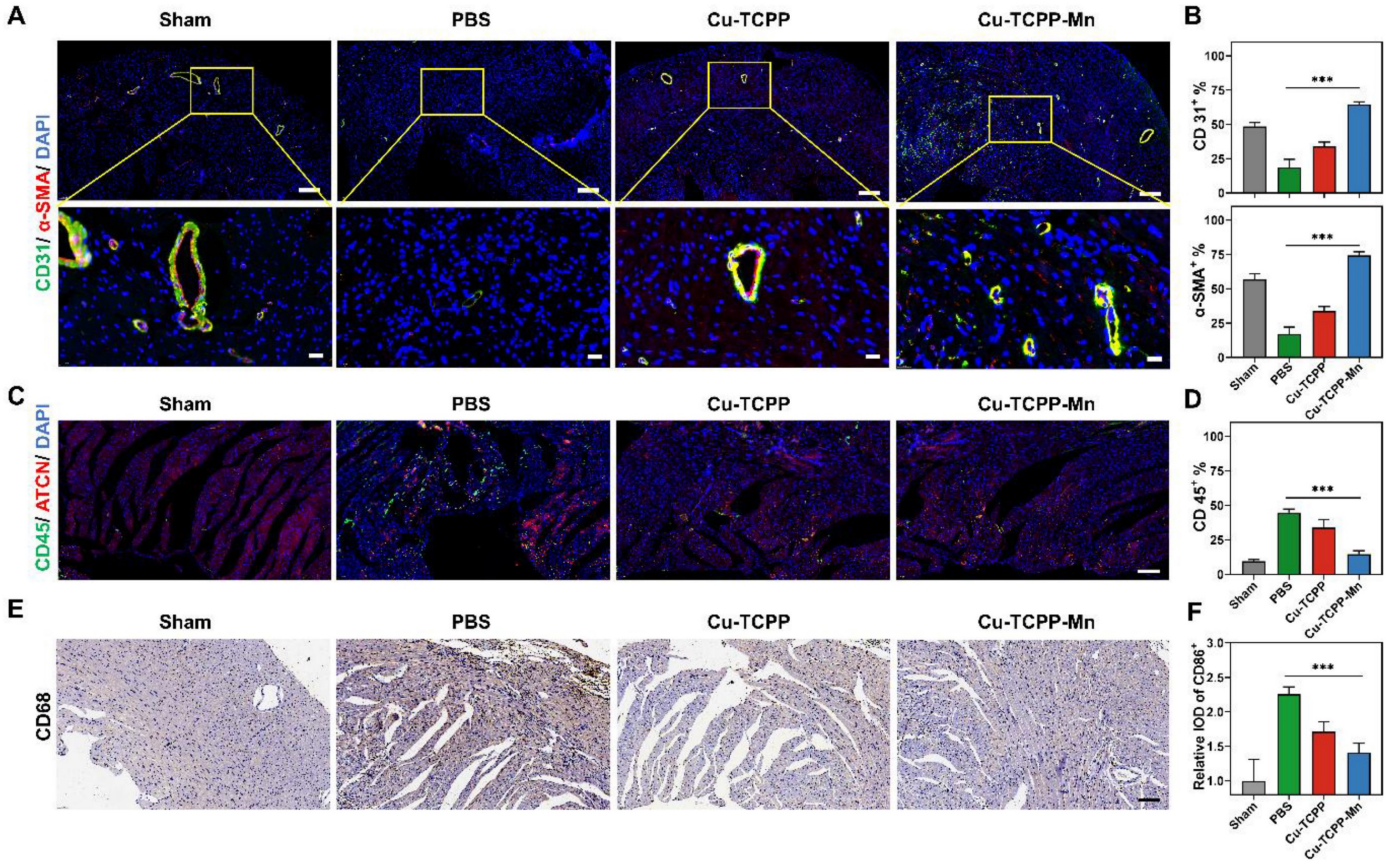
** Anti-inflammatory and angiogenesis activity of Cu-TCPP-Mn nanozyme in MI mice.** (A) Representative immunofluorescence images and (B) quantitative analysis of heart tissues co-stained with CD31 (vessels, green), α-SMA (α-smooth muscle actin, red), and DAPI (cell nucleus, blue) in Sham and MI mice treated with PBS, Cu-TCPP, and Cu-TCPP-Mn. Scale bar, 200 μm (top), 20 μm (bottom). (C) Representative immunofluorescence images and (D) quantification analysis of heart tissues co-stained with CD45 (neutrophil cell, green), ACTN (Actinin, red), DAPI (cell nucleus, bule) in Sham and MI mice treated with PBS, Cu-TCPP, and Cu-TCPP-Mn. Scale bar, 100 μm. (E) Representative heart tissue sections stained with CD68 (brown) and DAPI (blue) (F) and quantification analysis in MI mice treated with PBS, Cu-TCPP, Cu-TCPP-Mn. Scale bar, 100 μm.

In addition, the pro-angiogenic effect of the nanozyme in the heart after 28 days of MI was also accessed by immunofluorescence staining. The Cu-TCPP-Mn group effectively promoted neovascularization compared with other groups, as presented by the increased number of CD31^+^ and α-SMA^+^ capillaries that were distributed at the infarcted border zones (Figure 6B). The anti-inflammation effect of Cu-TCPP-Mn was determined by evaluating the inflammatory cells and expression of inflammatory biomarkers. As shown in Figure 6C-F, the infiltration of neutrophil granulocyte (CD45^+^) and macrophages (CD68^+^) in Cu-TCPP-Mn group was markedly decreased compared with the control group. Moreover, the pro-inflammatory cytokines of IL-1β and TNF-α in the serum were obviously decreased in the Cu-TCPP-Mn groups compared with other groups, while the anti-inflammatory cytokines of IL-10 in the serum were increased due to the anti-inflammation effect of Cu-TCPP-Mn (Figure S23). Afterwards, we analyzed the long-term toxicity of Cu-TCPP-Mn nanozyme *in vivo*. The mice were sacrificed 28 days post-treatment for biocompatibility evaluation. Major organs including kidney, liver, spleen and lung were harvested and stained with hematoxylin and eosin (H&E) (Figure S24). No apparent systemic toxicity was observed from the treated mice. Meanwhile, blood biochemistry assay of major liver and kidney damage biomarkers on mouse models after 28 days of treatment were tested, including alanine transaminase (ALT), aspartate transaminase (AST), alkaline phosphatase (ALP), blood urea nitrogen (BUN), creatinine (CRE) (Figure S25). There was a negligible difference in those damage biomarkers among different groups, suggesting the biosafety of Cu-TCPP-Mn nanozyme at the given dosage. Collectively, the above results demonstrated the anti-ROS and antioxidant function provided by Cu-TCPP-Mn nanozyme, leading to its myocardial protective effect.

### In vivo anti-inflammation on cardiac I/R injury rat models

**Figure 7:**
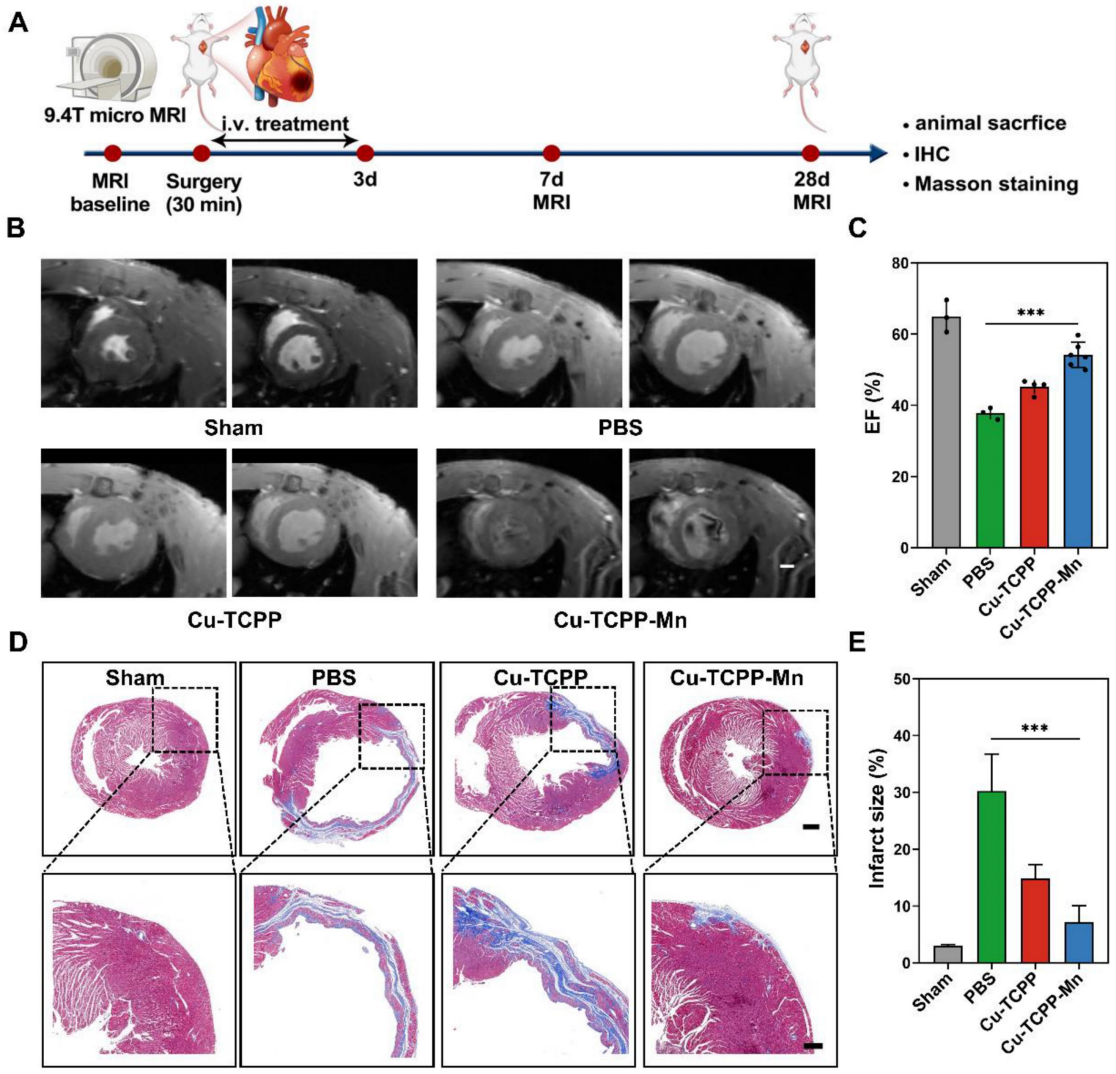
***In vivo* anti-inflammation of Cu-TCPP-Mn nanozyme on cardiac I/R injury rat models.** (A) The schematic of the study design. (B) Representative cine images of infarcted hearts in systolic and diastolic period collected from I/R injury rats scanned by a 9.4 T MRI. (C) EF calculated from 9.4 T MRI images after one week of treatments (n = 3-6 per group). (D) Representative Masson's trichrome-stained and (E) quantitative analysis of infarcted hearts in SD rats 4 weeks after treatment with PBS, Cu-TCPP, and Cu-TCPP-Mn. Data are presented as mean ± S.D. The comparisons between samples were operated by one-way ANOVA. * indicates P < 0.05; ** indicates P < 0.01; *** indicates P < 0.001; ns indicates P > 0.05 with no significance.

Reperfusion of the ischemic myocardium would induce excess production of ROS, resulting in myocardial dysfunction 69. In this project, we then further evaluated the therapeutic efficacy of Cu-TCPP-Mn nanozyme in cardiac I/R injury rat models. As shown in the schematic design, the rat models were established by surgical occlusion of the coronary artery for 30 min in 4 weeks male Sprague-Dawley (SD) rats, followed by 24 h reperfusion (Figure 7A) 64,70. I/R injury rats were administrated with PBS, Cu-TCPP, or Cu-TCPP-Mn with a dosage of 1.0 mg/kg for three times consecutively. 9.4 T micro magnetic resonance imaging (MRI) scanning was performed to monitor the cardiac function of rat models at day 7 and 28 71. The myocardial function was remarkably recovered when treated with Cu-TCPP-Mn, as confirmed by the increased EF calculated from the cine images (Figure 7B-C). In addition, Cu-TCPP-Mn nanozyme ameliorated myocardial fibrosis with the infarct size reduced from 30.7% ± 5.76% to 6.74% ± 2.69% (Figure 7D-E). To further explore whether Cu-TCPP-Mn could facilitate the myocardial repair together with cardiac function improvement, we stained heart tissues with CD45, CD68 and CD206. The IHC results revealed that Cu-TCPP-Mn could alleviate inflammation. In addition, heart tissues stained with CD31, α-SMA showed obvious increase of capillary density after Cu-TCPP-Mn treatment, as compared with other groups (Figure S26), indicating that Cu-TCPP-Mn enabled long-term angiomyogenesis, ventricular remodeling and heart function recovery. Histological evaluation of major organs in I/R injury rats was performed to evaluate the biosafety of Cu-TCPP-Mn nanozyme. No obvious tissue necrosis or adverse events were observed, indicating that Cu-TCPP-Mn nanozyme presented satisfactory histocompatibility (Figure S27). All these results fully confirmed the highly promising application potential of Cu-TCPP-Mn nanozyme for the treatment of cardiac I/R injury.

## Discussion

In summary, based on a bioinspired de novo design, we have developed a bimetallic MOF nanozyme to mimic cascade SOD and CAT activities for ROS elimination. The integrated Cu-TCPP-Mn nanozyme could be produced with a facile and simplified approach. It contains abundant Cu-active and Mn-active sites, which are endowed with high catalytic efficiency. As demonstrated by kinetic analysis and oxygen-production velocities analysis, Cu-TCPP-Mn nanozyme possesses good performance in both SOD- and CAT-like activities. *In vitro* experiments showed that the bimetallic MOF nanozyme has excellent ROS-scavenging activity and satisfactory biocompatibility. We established MI mouse models and cardiac I/R injury rat models to evaluate the anti-ROS and anti-inflammation ability of the Cu-TCPP-Mn nanozyme *in vivo*. To our delight, the bimetallic MOF nanozyme displayed superior performance in ROS scavenging and inflammation inhibition in the early inflammatory stage of cardiac injury, as compared with single-metallic MOF nanozyme. Moreover, it also showed long-term cardiac protection ability by remodeling ventricular structure, reducing scarring, promoting angiomyogenesis, and finally improving cardiac function. Overall, our study provides a feasible and applicable alternative to myocardial repair after MI and cardiac I/R injuries using bimetallic MOF nanozymes.

## Supplementary Material

Supplementary materials and methods, figures and tables.
