# Supplementary material for: MOF-derived bimetallic nanozyme to catalyze ROS scavenging for protection of myocardial injury
Source: Theranostics. 2023 Apr 29;13(8):2721–33. doi: 10.7150/thno.83543 (PMC10196836; doi:10.7150/thno.83543)
Supplement: Supplementary file 1 — Supplementary materials and methods, figures and tables. [file thnov13p2721s1.pdf]

Supporting Information

MOF-derived bimetallic nanozyme to catalyze ROS scavenging for protection of myocardial injury

Kaiyan Xiang[1#], Haoguang Wu[1#], Yu Liu[1#], Sheng Wang[2], Xueling Li[3], Bowei Yang[4,5], Yunming Zhang[1], Long Ma[1], Guangming Lu[1,2], Liangcan He[6*], Qianqian Ni[4,5,7*], Longjiang Zhang[1,2]*

Materials and Methods

Reagents and materials

Copper nitrate trihydrate ($Cu(NO_3)_2{\cdot}3H_2O$, 99%), 4,4,4,4-(Porphrine-5,10,15,20-tetrayl) tetrakis (benzoic acid) (H_2TCPP), N,N-dimethylformamide (DMF, 99.8%), benzoic acid (BA, 99.5%), Nitrotetrazolium blue chloride (NBT, CAS: 298-83-9), xanthine (X, CAS: 69-89-6), xanthine oxidase(XO, CAS: 9002-17-9), dihydroethidium (DHE, CAS:104821-25-2), Tris(4,7-diphenyl-1,10-phenanthroline) ruthenium(II) dichloride ($[(Ru(DPP)_3)]Cl_2$, CAS: 207802-45-7), 2',7'- Dichlorofluorescin Diacetate (DCFH-DA, ≥97%, CAS: 4091-99-0), Thiazolyl Blue Tetrazolium Bromide (MTT), 2,3,5-Triphenyl tetrazolium chloride (TTC, 98.0%) were purchased from Sigma-Aldrich (Shanghai, China). Manganese (II) chloride tetrahydrate ($MnCl_2{\cdot}4H_2O$) was bought from Shanghai Qingxi Chemical Technology Co. Ltd. (Shanghai, China). Cy5-NHS ester (CAS: 146368-14-1) was bought from Meilunstar, Dalian Bergolin Biotechnology Co., Ltd (Liaoning, China). Tris-HCl buffer (1 M, pH 6.8), Tris-HCl buffer (1 M, pH 8.8) and Hydrogen peroxide assay kit were obtained from Beyotime

Chemical Reagent Co. Ltd. (Jiangsu, China). Antifade mounting medium with DAPI was obtained from VECTASHIELD (Vector laboratories, Inc, USA). Hoechst 33342 dyes were obtained from Invitrogen (Thermo Fisher Scientific, USA). All commercially available chemicals were analytical grade and used without further purification.

Measurements and characterizations:

Morphology of the synthesized Cu-TCPP and Cu-TCPP-Mn nanozymes were characterized by JEM-2100 F transmission electron microscopy (TEM, JEOL, Japan) at an acceleration voltage of 200 kV and a Dimension ICOM atomic force microscopy (AFM, Bruker, Germany). Scanning electron microscope (SEM) images were collected on a Zeiss sigma 500 microscope (Zeiss, Germany). Fourier transform infrared (FT-IR) spectrum was carried out with a Nicolet IS5 FTIR spectrometer (Thermo, USA). X-ray diffraction (XRD) patterns were recorded on a Smartlab 9 kW X-ray diffractometer (Rigaku, Japan) in the 2θ range of 5-90° at a speed of 10°/min. X-ray photoelectron spectroscopy (XPS) was collected on the PHI5000 VersaProbe spectrometer (ULVAC-PHI, Japan). UV-visible absorption spectra were recorded using a UV-visible spectrophotometer (PerkinElmer, USA). Zeta potential and hydrated particle size were performed on a Brookhaven dynamic light scattering (DLS). The amount of copper (Cu) and manganese (Mn) in nanozyme was analyzed by an inductively coupled plasma atomic emission spectrometer (ICP-AES) (Avio 500, PerkinElmer,). The generated dissolved oxygen (milligram per liter) was detected by an Orion Star A323 RDO/Dissolved oxygen portable meter (Thermo, USA). Biomarkers (LDH, LDH1, AST, ALT, ALP, CREA, BUN) were detected by fully automatic biochemical analyser (Chemray 240, Shenzhen). The

fluorescence images were taken by laser scanning confocal fluorescence microscopy (LSCFM, Leica TCS SP8, Shanghai). The fluorescence images of Cy5-Cu-TCPP-Mn *ex vivo* biodistribution were captured by In-vivo Optical Imager (InVivo Smart-LF, VISQUE, Korea). Echocardiography was performed by a Vevo2100 digital imaging system (Visual Sonics Toronto, ON, Canada). Cardiac MRI examinations were performed with a 9.4 T MR animal scanner (94/30 USR, Bruker, Germany) equipped with synchronous acquisition system of ECG.

Synthesis of Cu-TCPP and Cu-TCPP-Mn nanozymes

Cu-TCPP nanosheets were synthesized according to the previous studies [1]. Firstly, 10.0 mL of 1.0 mmol/L H_2TCPP dissolved in N, N-dimethylformamide (DMF) and 5.0 mL of 10 mM $Cu(NO_3)_2$ dissolved in deionized water were mixed in a 500 mL round-bottomed flask. Then, 0.8 g benzoic acid together with 10 mL DMF was added to the flask to facilitate the formation of homogeneous solution. The mixed solution was reacted for 4 h in methyl silicone oil at 90 ℃ under stirring. The Cu-TCPP products were then washed with DMF or ethanol for three times and obtained by ultracentrifugation at 12000 rpm for 10 min.

Cu-TCPP-Mn nanosheets were further prepared by mixing the obtained Cu-TCPP products with 10 mL $MnCl_2$ (1mM). The mixed solution was stirred (300 rpm) and heated at 90 ℃, and then refluxed for 12 h overnight. After cooling down to room temperature, the purple resultant precipitate can be collected by centrifugation and washed with DMF or ethanol for three times. Then, the Cu-TCPP-Mn nanosheets solution was sonicated for 6 h at 300 W with an ultrasound probe. The resulting solution was centrifuged for 10 min at 13000 rpm and washed

with deionized (DI) water for three times. Finally, the supernatant containing Cu-TCPP-Mn nanodots was obtained.

Detection of SOD-like activity of Cu-TCPP-Mn

As a sensitive $^{\cdot}O_2^-$ probe, nitrotetrazolium blue chloride (NBT) was applied to measure the SOD activities of Cu-TCPP-Mn and Cu-TCPP. Briefly, different concentrations of Cu-TCPP-Mn and Cu-TCPP nanozymes were mixed with X (0.15 mM) and XO (0.02 U/mL) in tris-HCl buffers with different pH values (0.1 M, pH 5.5, 6.8 and 8.8) at 37 °C and 20 °C, respectively. After 5 min of co-incubation, NBT (0.05 mM) was added to each mixed solution in a microplate reader. The absorbance was set at 550 nm and detected every 15 s continuously *via* a UV-vis spectrophotometer for 5 min.

Similarly, dihydroethidium (DHE) was used to assess the SOD-like activities of Cu-TCPP-Mn nanozymes. DHE has been used for ROS detection as a superoxide fluorescence probe by measuring the amount of $^{\cdot}O_2^-$. First, X (0.6 mM), XO (0.05 U/mL) and different concentrations of Cu-TCPP-Mn/Cu-TCPP nanozymes were mixed in tris-HCl buffer at 37 °C for 15 min. DHE (0.5 mg/mL) was then added to the mixed solution for another 15 min. After the reaction, the fluorescence spectra were used to measure the fluorescence intensities of mixed solutions (excitation: 470 nm, emission: 610 nm).

Detection of CAT-like activity of Cu-TCPP-Mn

CAT-like activities of Cu-TCPP-Mn and Cu-TCPP nanozymes were evaluated by $[Ru(dpp)_3]Cl_2$, which was a luminescent oxygen sensor. Typically, a pathological concentration of 100

μM H_2O_2 and different concentrations of nanozymes were mixed together in tris-HCl buffer (0.1 M). Then, [Ru(dpp)$_3$] Cl_2 (10 mg/mL, 10 μL) was added to detect the generation of O_2 at 460 nm.

Additionally, a dissolved-oxygen portable meter was used to monitor the generation of oxygen directly. Typically, 30, 60, 90, 120,150 and 200 μM H_2O_2 was mixed with different concentrations of nanozymes (1 mL) in 15 mL of tris-HCl buffer with different pH (0.1 M, pH 5.5, 6.8 and 8.8) at 20 °C and 37 °C. Then, the production of oxygen was detected by using a dissolved-oxygen portable meter in 10 min.

Kinetic analysis

The NBT kinetic assay of SOD-like activities measured at 550 nm using a UV-vis spectrophotometer was recorded and imported in GraphPad Prism 8.0 to analyze enzyme kinetic data. The inhibition rate was calculated by the formula: Inhibition rate% = $(Absorbance_{blankA}$- $Absorbance_{sample})$/ $(Absorbance_{blankA}$- $Absorbance_{blankA0})$ × 100%. As for the kinetic analysis of CAT-like activities of Cu-TCPP and Cu-TCPP-Mn, the generation of dissolved O_2 under different conditions was measured and recorded at different time points. The Michaelis-Menten constant Km and Vmax were also automatically calculated by GraphPad Prism 8.0 software.

Cytotoxicity assay

RAW264.7, bEnd.3 or H9C2 cells were cultured in high-glucose Dulbecco's modified Eagle's medium (DMEM), which contained 1% penicillin-streptomycin (10000 U/mL) and 5% fetal

bovine serum. Cells were then seeded with a density of 1 × 10^4 per well in 96-well plates for 24 h at 37 °C. Different concentrations (5, 10, 20, 50 μg/mL) of Cu-TCPP and Cu-TCPP-Mn nanozymes were added in cells for another 24 h separately. Afterwards, the cells were gently washed by PBS (pH 7.4) for three times. 5 μg/mL Thiazolyl Blue Tetrazolium Bromide (MTT) was added into the 96-well plates for another 4 h in the dark. The cell supernatant was pipetted by micropipettor and 150 μL DMSO was added to the cell supernatant. After horizontal jitter for 10 min to fully dissolved the crystallization of MTT, cell viability was determined by a microplate reader at the absorbance of 490 and 570 nm.

Hemolysis assay

The fresh blood sample (1 mL) was collected from healthy C57BL/6 mouse. The blood sample was centrifugated at 2000 rpm for 5 min to remove the upper serum and obtain red blood cells (RBCs). The RBCs were further washed with 0.9% saline for three times and diluted to 2% RBC suspension. Then, different concentrations of Cu-TCPP-Mn (0.8 mL, 1-1000 μg/mL sample), 0.9% saline (0.8 mL, negative control), or DI water (0.8mL, positive control) were added to 0.2 mL RBC suspension, respectively. All sample tubes were kept at room temperature for 2 h and then centrifuged at 2000 rpm for 5 min. The supernatant of all sample tubes was transferred into a 96-well plate for detection (n = 4). The absorbance value was measured at 545 nm. Finally, the hemolysis rate was calculated according to the formula: Hemolysis rate (%) = $(A_{sample} - A_{negative\ control})/(A_{positive\ control} - A_{negative\ control}) * 100\%$ [2].

Cell apoptosis analysis by flow cytometry.

H9C2 cells were seeded in confocal dishes and then incubated with different concentrations of enzyme mimics (1, 2.5, 5 μg/mL) for 4 h at 37 °C. The cells were then incubated at 37 °C for 4 h with H_2O_2 (100 μM), and stained with 500 nM propidium iodide (PI) for 15 min. The treated cells were washed gently by PBS for three times and collected by centrifugation. Then, the fluorescence intensity of all treated cells was assessed by a flow cytometer.

Subcellular Location of Cu-TCPP-Mn

The subcellular location of Cu-TCPP-Mn nanozymes was investigated by LSCFM. First, 5×10^5 H9C2 cells were plated on 10 mm confocal petri dishes and incubated for 12 h. Then, 50 μg/mL Cy5 labeled Cu-TCPP-Mn were incubated for 4 h. Afterwards, the cells were stained with MitoTracker Green(50 nM) or LysoTracker Green(50 nM) at 37 °C for 30 min and then washed gently by PBS and stained with 1 μg/mL Hoechst 33342 for 5 min. Then, LSCFM was used to observe the fluorescence of cells with Ex/Em 350/461 nm for Hoechst33342 (blue) and Ex/Em 490/511 nm for either MitoTracker Green or LysoTracker Green.

Evaluation of intracellular ROS-scavenging ability by LSCFM and flow cytometry

The intracellular levels of ROS were monitored by using the fluorescent probe of H_2DCFH-DA, which reacts with intracellular free radicals and produces a fluorescent product, dichlorofluorescein (DCF). The excitation and emission wavelengths of the fluorescent product were 488 and 525 nm, respectively. RAW264.7 cells were planked in 10 mm confocal petri dishes with a density of 5×10^5 cells per well for 24 h at 37 °C. Then, 1 μg/mL LPS (Rosup) and 5 μg/mL of nanozymes were added to each well for another 24 h. After being

gently washed by PBS (pH 7.4) in triplicate, 10 μM H_2DCFH-DA was added to RAW264.7 cells followed by 30 min incubation in the dark. After 30 min, cells were gently washed by fresh medium. 1 μg/mL Hoechst 33342 dye were added and incubated for another 5 min. Then, LSCFM was used to observe the fluorescence of RAW264.7 cells. Similar protocol was applied to flow cytometry experiment for DCF fluorescence detection expect for trypsin digestion of cells followed by DCFH-DA staining.

***In vivo* biodistribution analysis**

Male C57BL/6 (6-8 weeks old, 20-25 g) mice which were subjected to cardiac thoracic surgery or sham surgery were divided into MI and non-MI (sham) groups, respectively. For *ex vivo* fluorescent imaging, 200 μL Cy5 labeled Cu-TCPP-Mn at the concentration of 1 mg/mL was injected into different groups of mice through the tail vein (n = 3). At 6 or 12 h post-injection, the healthy or MI hearts and other major organs (liver, kidney, lung and spleen) were harvested for *ex vivo* fluorescent imaging. The mean fluorescence intensities of the isolated organs from each group were recorded and analyzed by GraphPad Prism 8.0 software. Then, as for detection of nanomaterials retention, two groups of mice were injected with Cu-TCPP-Mn solutions (25 mg/kg) through the tail vein (n = 3). At 24 h post-injection, the key organs (heart, lung, liver, kidney, spleen) from all treated groups were harvested and weighed, after which ICP-AES was performed for detection of the Cu contents to figure out the retained conditions of nanomaterials in different organs.

***In vivo* assessment in acute MI models**

All of our animal studies were approved by the Committee for Experimental Animals Welfare and Ethics of **Jinling Hospital**, Medical School of Nanjing University. Male C57BL/6 (6-8 weeks old, 20-25 g) mice were subjected to cardiac thoracic surgery as permanent ligation of the left anterior descending (LAD) coronary, or sham surgery (thoracotomy without ligation) [47,48]. Mice were anesthetized initially with 4% isoflurane inhalation followed by 1% isoflurane inhalation for anesthesia maintenance, and ventilated on a rodent respirator via a tracheostomy. A 1.5-cm incision was made between the third and fourth ribs in lower-left sternum, and the pectoral major and pectoral minor muscles were separated to expose the fourth intercostal space. After exposing the heart through thoracic cavity, the LAD artery was ligated 2 mm lower from the left auricle with a 7-0 silk thread. Quick reset of the heart back into the thoracic cavity and gently evacuating the air out of the chest cavity manually were needed. Successful LAD ligation was confirmed by visually observing a pallor of the anterior wall of the left ventricle and by the presence of an ST-segment elevation on an electrocardiogram. After 15-min recovery from the surgery, Cu-TCPP (0.5 mg/mL), Cu-TCPP-Mn (0.5 mg/mL) or PBS (200 μL) were injected through tail vein, respectively. The treatments were given once every day for three times. At different time points after MI surgery, cardiac function and ventricular structure were monitored by echocardiography (VisualSonics Vevo 2100 imaging system) which provided measures of heart rate (HR), left ventricular ejection fraction (LVEF), left ventricular fractional shortening (LVFS), left ventricular fractional area change (LVFAC), left ventricular anterior wall thickness (LVAW), left ventricular posterior wall thickness (LVPW), left ventricular volume and LV mass[s(systolic)/d(diastolic)]. After the last time point of echocardiography, all groups of

mice were euthanized for following evaluation of therapeutic effect.

Determination of myocardial injury by LDH release

Serum LDH levels represent the major biomarkers of ischemic severity. 3 days post treatment of nanozyme injection, the blood serum of each group was collected for biochemical parameter analysis (Fully automatic biochemical analyzer, Chemray 240, Shenzhen) to detect the levels of LDH and LDH1. Quantitative analysis of each group was evaluated by GraphPad Prism 8.0 software.

Evaluation of anti-inflammation in MI mice

The evaluation of therapeutic efficacy of Cu-TCPP-Mn nanozyme on MI mice was mainly operated by histological study. Frozen sections of hearts tissue from each group were selected and cut into cross sections for ROS staining (DHE and DCFH-DA, respectively). The ROS fluorescence image of each heart tissue was photographed by CLSFM using the same parameter. Quantitative analysis for the ROS fluorescent intensity was determined by ImageJ software. Three of remaining heart tissues from each group were paraffin-embedded and cut into cross sections for Masson's trichrome staining and immunofluorescent staining for TUNEL, myeloid cell markers (CD45), angiogenesis markers (α-SMA, CD31). Quantitative analysis for the collagen contents and each positive marker of heart tissues was operated by ImageJ software.

Infarct size evaluation

3 days post nanozyme injection, three fresh hearts of each group were excised and frozen in -80 ℃ refrigerator for 20 min. The hearts were dissected into 2 mm slices and stained with 2% triphenyltetrazolium chloride (TTC) for 20 min at 37 ℃ in the dark. After washing with PBS, heart slices were then imaged and weighed. Infarct size was represented by white region whereas viable tissues were represented by red region. The quantitative analysis of the infarct area was calculated by ImageJ software.

***In vivo* assessment of rat myocardial I/R model**

For the myocardial I/R models, the surgery was performed as previously described [49]. Male Sprague-Dawley (SD) (4-6 weeks old, 200-300 g) rats were subjected to cardiac thoracic surgery as 30-min temporary ligation of the left anterior descending (LAD) coronary, or sham surgery (thoracotomy without ligation) under anesthesia of isoflurane and ventilation of rodent respirator. The rats performed with temporary ligation of LAD coronary were loosened for reperfusion for 12 h. Then I/R rats were randomized into 3 groups treated with PBS (500 μL), Cu-TCPP (1.0 mg/mL) and Cu-TCPP-Mn (1.0 mg/mL), respectively through tail vein. The treatments were performed once every day for 3 times. After treatments, all rats were performed with cardiac MR imaging on a 9.4 T micro MR scanner, which was combined the ECG trigger with a respiration signal to start data acquisition. T_1-weighted MR imaging and cine imaging was performed before (baseline) and at 7 day and 28 day post injection to observe the change of cardiac morphology and function. The T1-weighted cardiac MRI parameters were used as follows: TR 104.169 ms, TE 2.500 ms, FOV 50 × 50 mm^2, flip angel 40°, slice thickness 1 mm, matrix 256 × 256. Cine FLASH cardiac MRI parameters were used

as follows: TR 8.000 ms, TE 1.600 ms, FOV 50 × 50 mm^2, flip angel 15°, slice thickness 1mm, matrix 192 × 192.

In vivo biosafety and toxicity evaluation

To evaluate the biosafety and toxicity of the nanozyme, healthy and I/R male SD rats were used. Different concentration of nanozyme or equal volume of 0.9% NaCl was administered by lateral cerebral ventricle injection. The rats were dissected, and the main organs (brain, heart, liver, spleen, lung and kidney) were harvested for HE staining.

Statistical Analysis

All data were presented as mean ± S.D. Data were compared by one-way ANOVA with Bonferroni correction for multiple-group comparisons. Significant difference between treatment and control groups: *indicates $P < 0.05$, **indicates $P < 0.01$, ***$P < 0.001$, ****$P < 0.0001$, ns indicates $P > 0.05$ with no significance.

Supplementary Figures and Tables

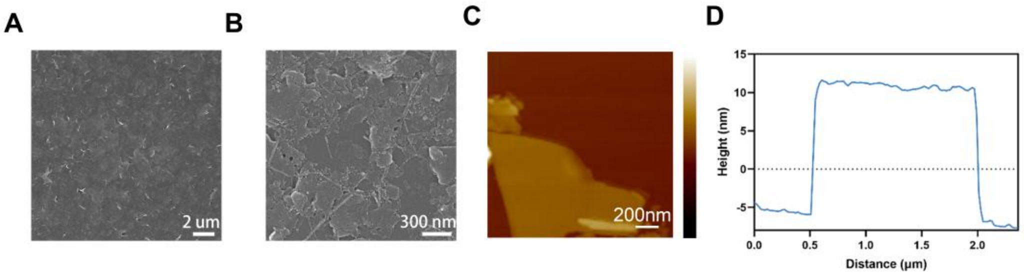

Figure S1. Characterization of Cu-TCPP nanosheets. (A) Representative TEM image of Cu-TCPP nanosheets. (B) Representative SEM image of Cu-TCPP nanosheets. (C) Representative AFM image and (D) thickness of Cu-TCPP nanosheets.

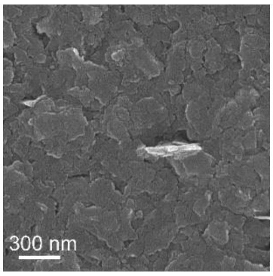

Figure S2. SEM image of Cu-TCPP-Mn nanosheets.

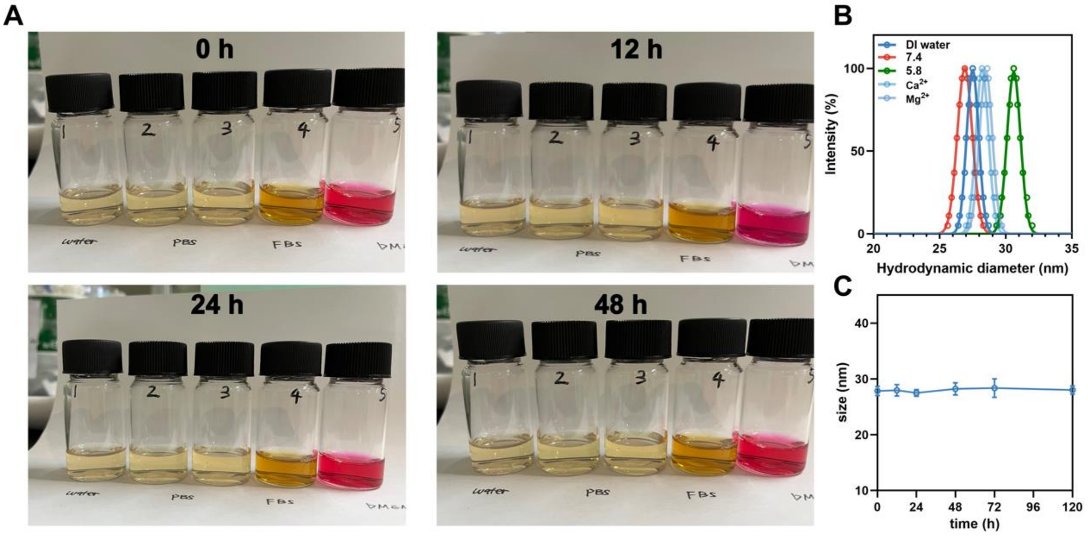

Figure S3. Stability assessment of Cu-TCPP-Mn nanozymes. (A) The photos of Cu-TCPP-Mn nanodots dispersed in absolute ethyl alcohol (# 1), deionized water (# 2), phosphate-buffered saline (PBS) (# 3), Fetal Bovine Serum (FBS) (# 4) and Dulbecco's modified Eagle's medium (DMEM) (# 5) at different time points. (B) Dynamic light scattering (DLS) analysis of Cu-TCPP-Mn nanozymes in deionized water. (C) DLS analysis of Cu-TCPP-Mn nanozymes in water at different time points.

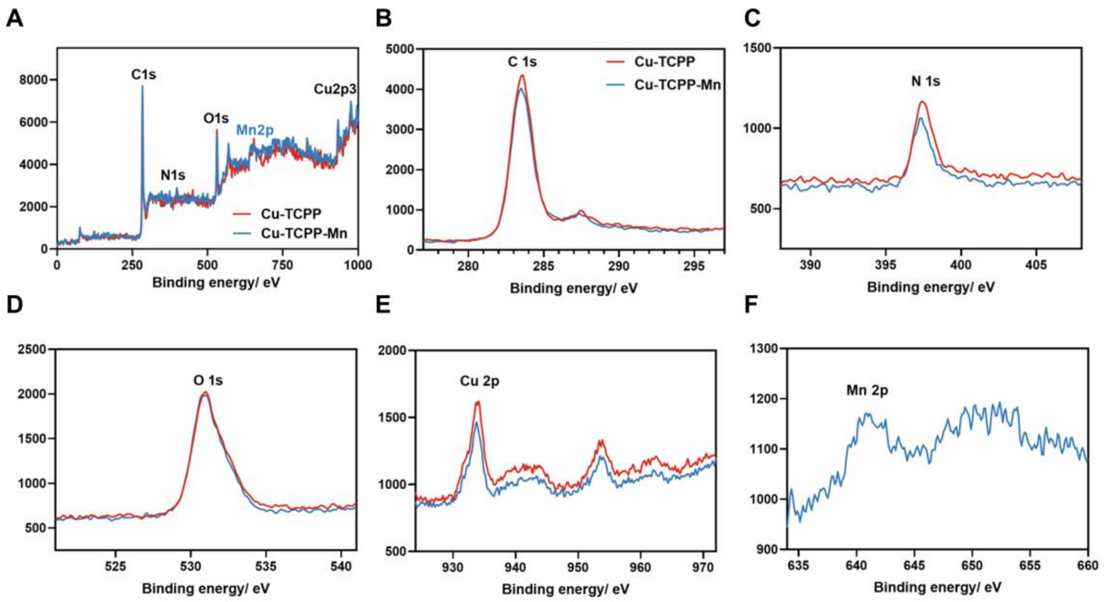

Figure S4. Characterizations of Cu-TCPP-Mn nanodots. (A) Full XPS, (B) C 1s, (C) N 1s, (D) O 1s, (E) Cu 2p, and (F) Mn 2p spectra of the prepared Cu-TCPP-Mn nanodots.

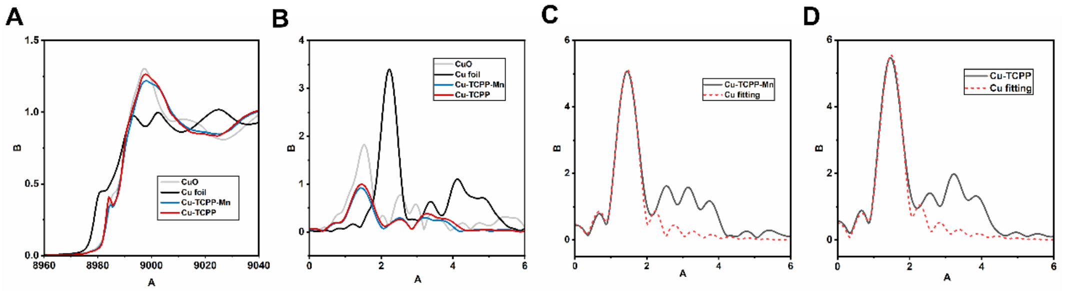

Figure S5. Cu K-edge XANES and EXAFS of Cu-TCPP and Cu-TCPP-Mn. (A) Normalized Cu K-edge XANES spectra of different samples. (B) Fourier transform EXAFS spectra (k^3-weighted). (C) Cu K-edge EXAFS of Cu-TCPP-Mn in the R space and the fitting curves without correcting for the scattering phase shift. (D) Cu K-edge EXAFS of Cu-TCPP in the R space and the fitting curves without correcting for the scattering phase shift.

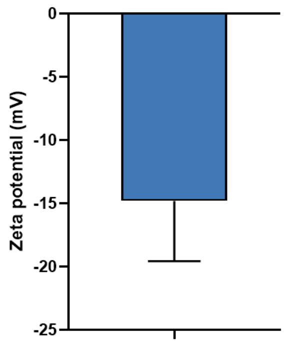

Figure S6. Zeta potential of Cu-TCPP-Mn nanodots.

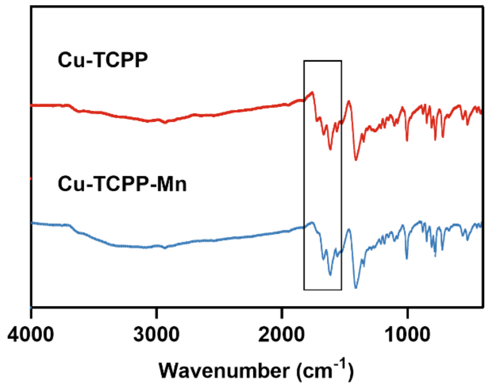

Figure S7. FTIR of Cu-TCPP-Mn nanodots.

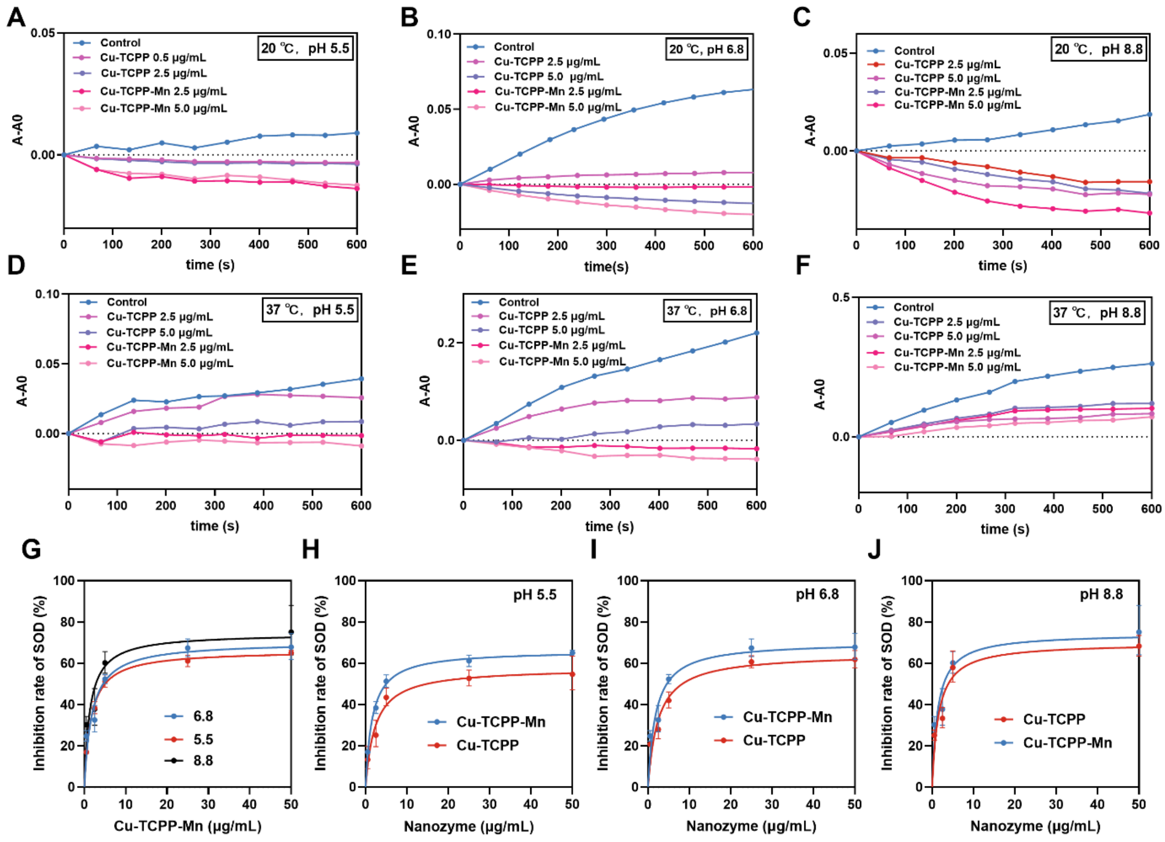

Figure S8. SOD-like activity detected by nitrotetrazolium blue chloride (NBT) assay. (A-F) Comparison of SOD-like activity of Cu-TCPP and Cu-TCPP-Mn under different pH (5.5, 6.8, 8.8) at 20 ℃ and at 37 ℃. (G) Inhibition rate curve of SOD-like activity of Cu-TCPP-Mn nanozyme. (H-J) Inhibition rate curves of Cu-TCPP-Mn under different pH conditions.

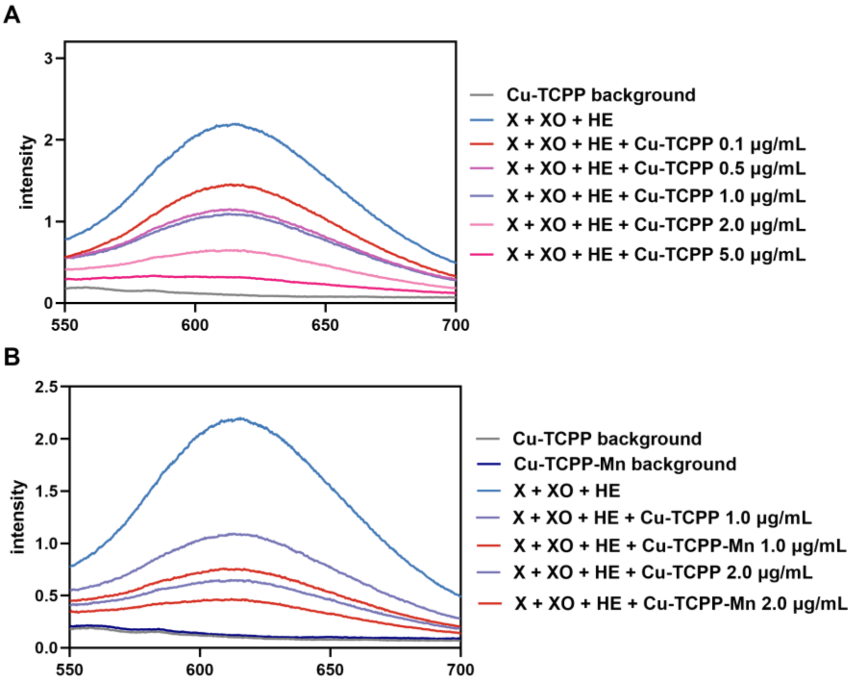

Figure S9. SOD-like activity of Cu-TCPP. (A) and Cu-TCPP-Mn (B) detected by dihydroethidium (DHE), a superoxide probe for ROS detection.

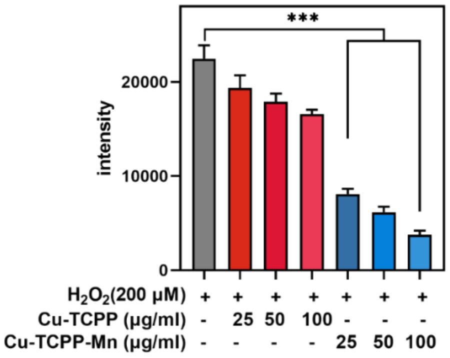

Figure S10. CAT-like activity of Cu-TCPP and Cu-TCPP-Mn by detection of H_2O_2 consumption using Ru(dpp)Cl_2.

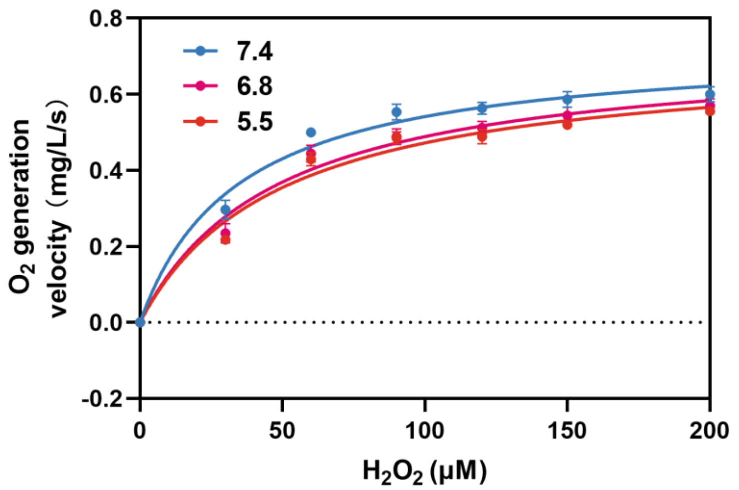

Figure S11. Kinetic curves of generated oxygen for CAT-like activity of Cu-TCPP-Mn under different pH (5.5, 6.8, 7.4) conditions.

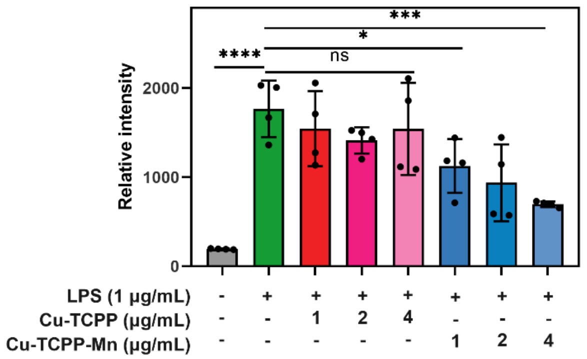

Figure S12. The ROS level of RAW 264.7 cells treated with Cu-TCPP (1, 2, 4 μg/mL) and Cu-TCPP-Mn (1, 2, 4 μg/mL) as detected by the spectrofluorimetry.

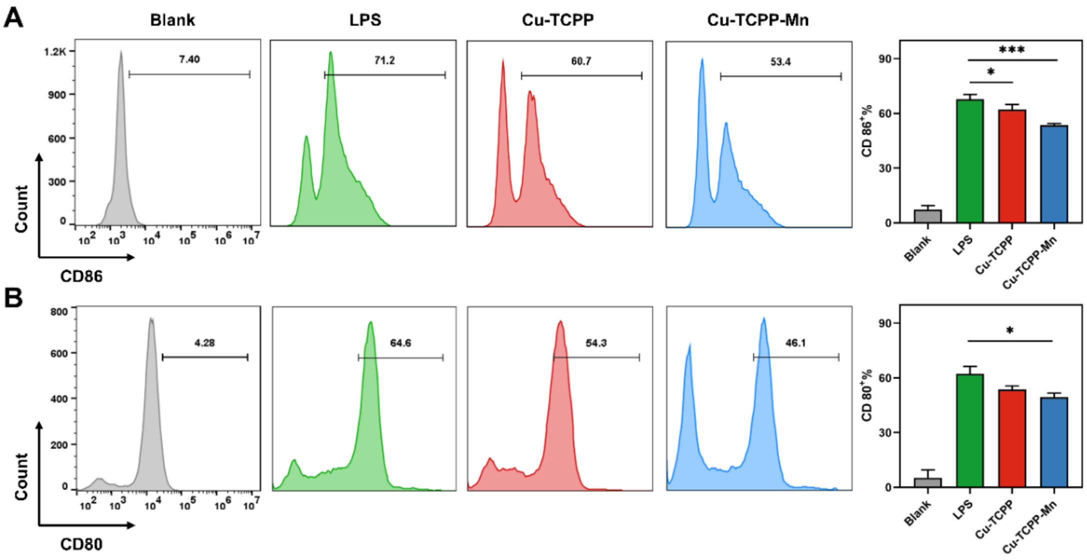

Figure S13. Flow cytometry and quantitative analysis of CD86 (A) and CD80 (B) expression in RAW 264.7 cells cultured under different conditions (Saline, LPS, LPS + Cu-TCPP, LPS + Cu-TCPP-Mn) for 12 h.

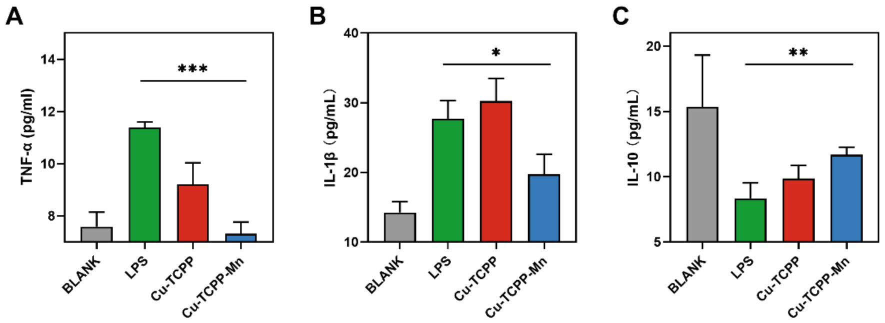

Figure S14. The levels of pro-inflammatory cytokines (IL-1β, TNF-α) and anti-inflammatory cytokines (IL-10) in cellular supernatants with different treatments.

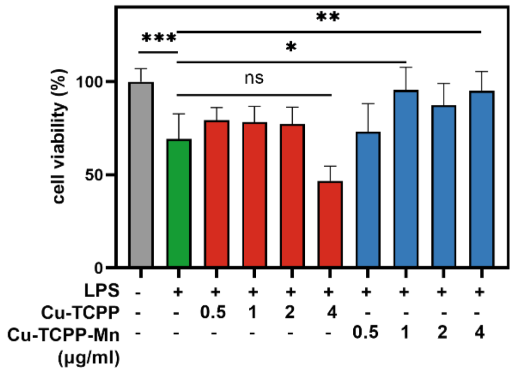

Figure S15. Cell viability of LPS stimulated RAW264.7 cells treated with Cu-TCPP (0.5, 1, 2, 4 μg/mL) and Cu-TCPP-Mn (0.5, 1, 2, 4 μg/mL).

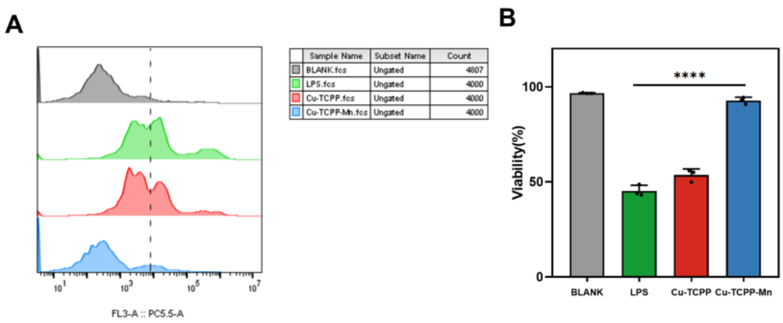

Figure S16. (A) Cell apoptosis of H9C2 cells treated with Cu-TCPP and Cu-TCPP-Mn nanozymes analyzed by flow cytometry. (B) Quantitative analysis of cell viability.

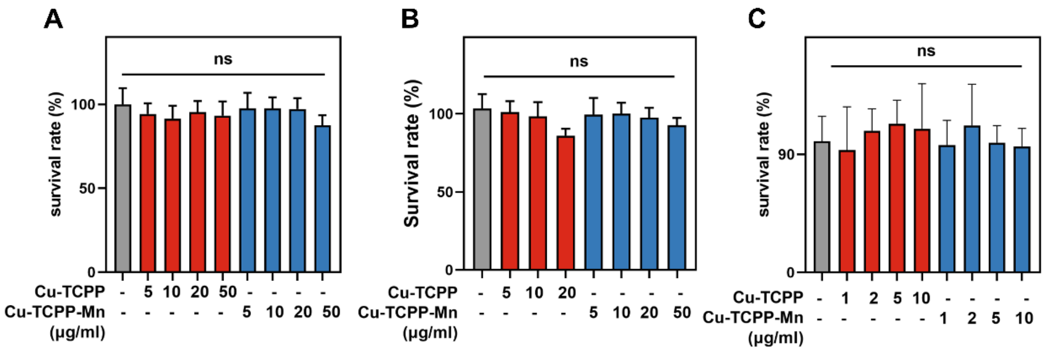

Figure S17. Cytotoxicity analysis of (A) H9C2, (B) bEnd.3 and (C) RAW264.7 cells treated with Cu-TCPP (5, 10, 20, 50 μg/mL) or Cu-TCPP-Mn (5, 10, 20, 50 μg/mL).

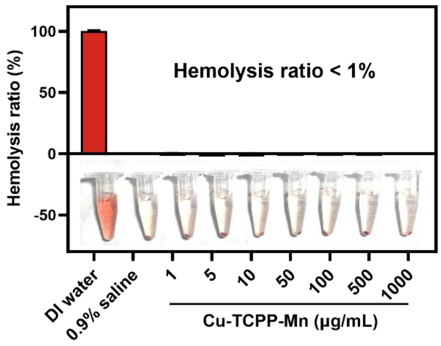

Figure S18. Hemolysis percentage of RBCs incubated with DDI water, saline and Cu-TCPP-Mn. Inset: Photos of hemolysis in different systems.

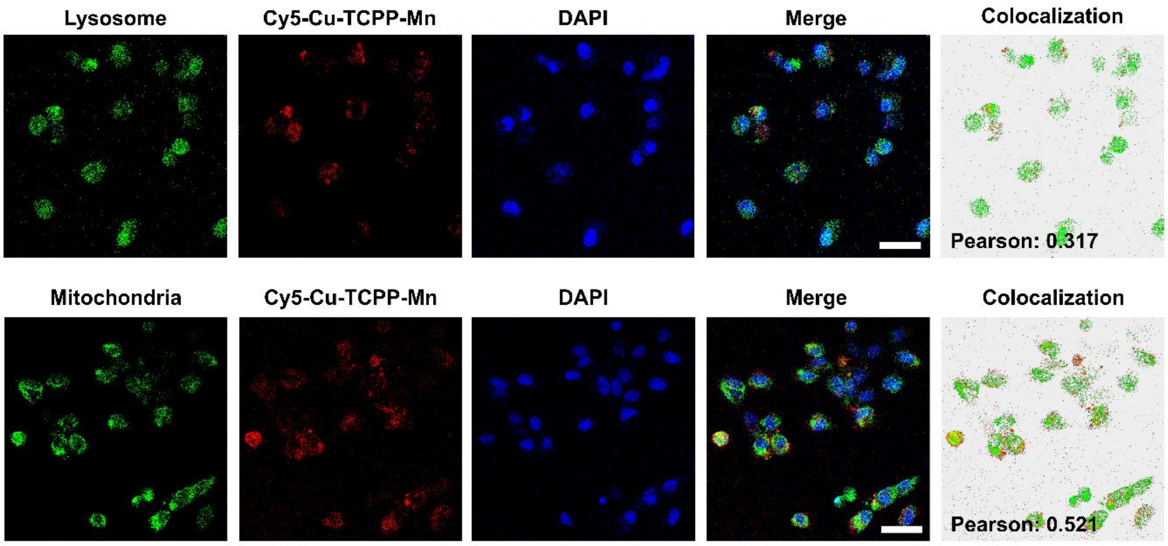

Figure S19. Representative confocal images of subcellular distribution of Cy5-labeled Cu-TCPP-Mn after uptake by H9C2 cells. Mitochondria was stained with commercial MitoTracker (Green), and lysosome was stained with commercial LysoTracker (Green), cell nucleus was stained with DAPI (blue). Scale bar: 50 μm. The Pearson's correlation coefficient of Cu-TCPP-Mn and mitochondria is 0.521, and the Pearson's correlation coefficient of Cu-TCPP-Mn and lysosome is 0.317.

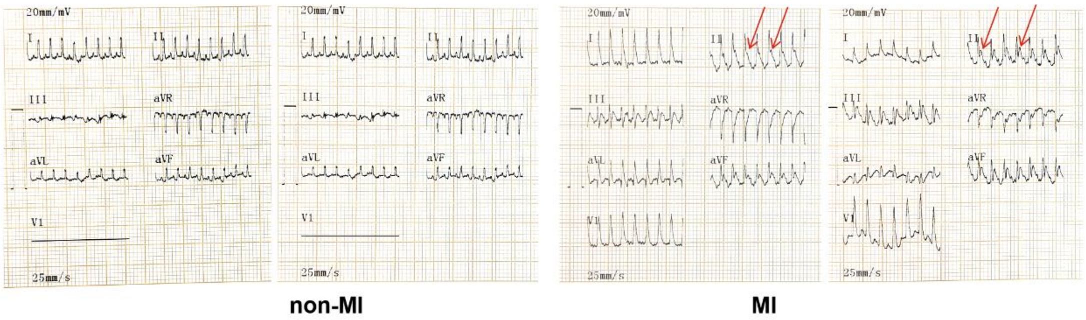

Figure S20. ECG images collected from non-MI (Sham) and MI mice. ST-segment elevation (red arrow) represented the successful ligation of LAD.

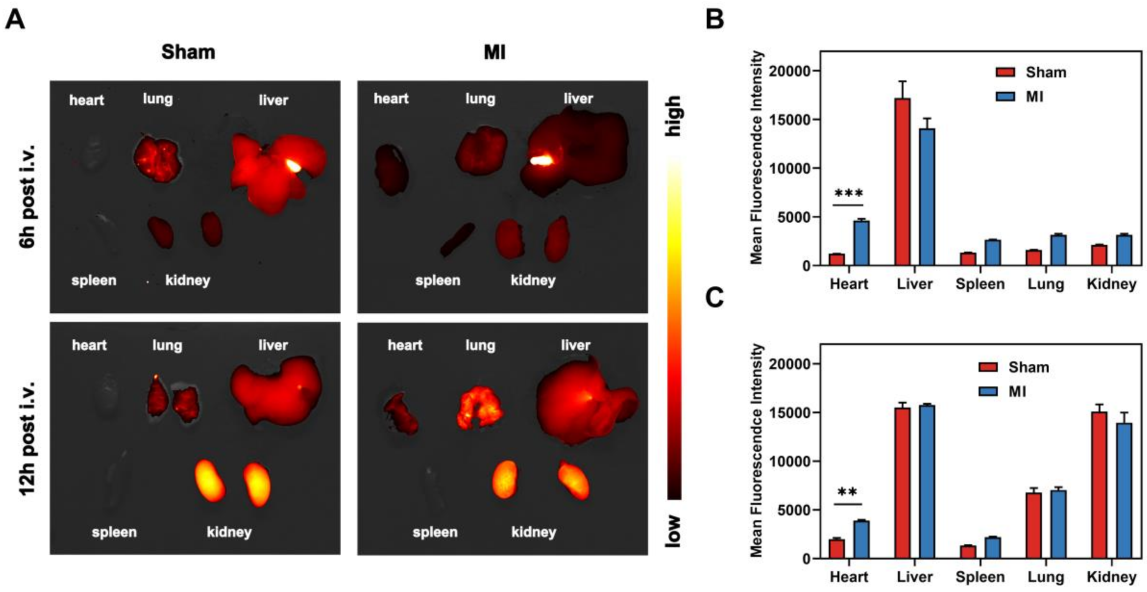

Figure S21. Biodistribution assay of Cu-TCPP-Mn. (A) Representative *ex vivo* fluorescence images of Cy5 labeled Cu-TCPP-Mn nanozyme in major organs harvested from sham and MI mice at 6h and 12h post intravenous injection (n = 3 animals per group). Mean fluorescence intensities of the isolated organs from each group at (B) 6 h post-injection and (C) 12 h post-injection.

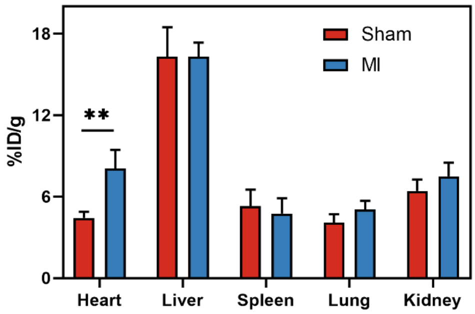

Figure S22. Biodistribution of Cu-TCPP-Mn in major organs of normal and MI mice at 24 h post injection (n = 4).

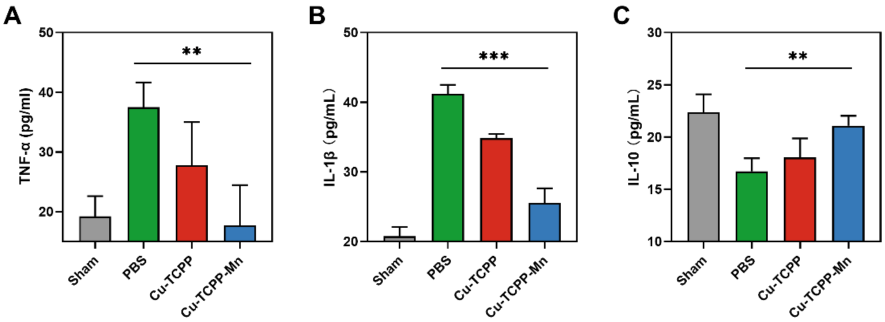

Figure S23. The serum levels of TNF-α (A), IL-1β (B) and IL-10 (C) from mice with different treatments detected by commercial ELISA kits.

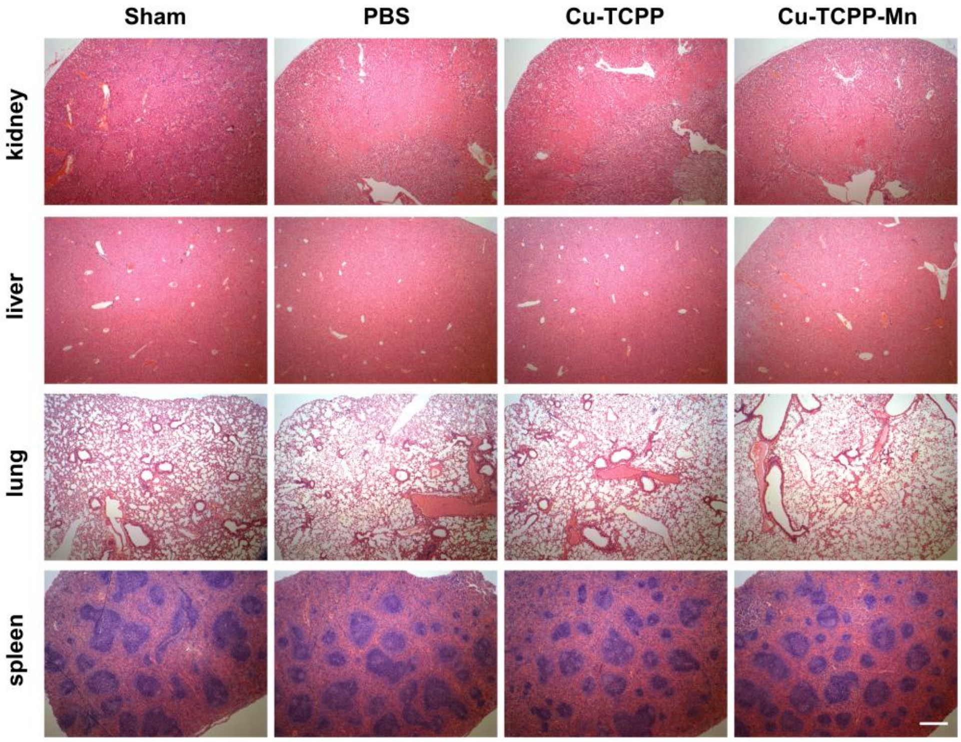

Figure S24. H&E staining of major organ tissues harvested from sham or MI mice models treated with Cu-TCPP and Cu-TCPP-Mn. Scale bar, 500 μm.

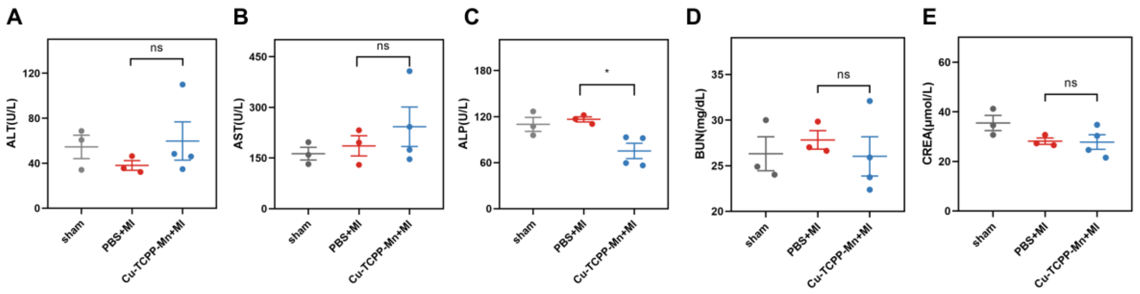

Figure S25. Blood analysis of MI mice models with the treatment of Cu-TCPP-Mn. (A) Alanine transaminase (ALT), (B) aspartate transaminase (AST), (C) alkaline phosphatase (ALP), (D) blood urea nitrogen (BUN), (E) creatinine (CREA) concentrations detected in the serum.

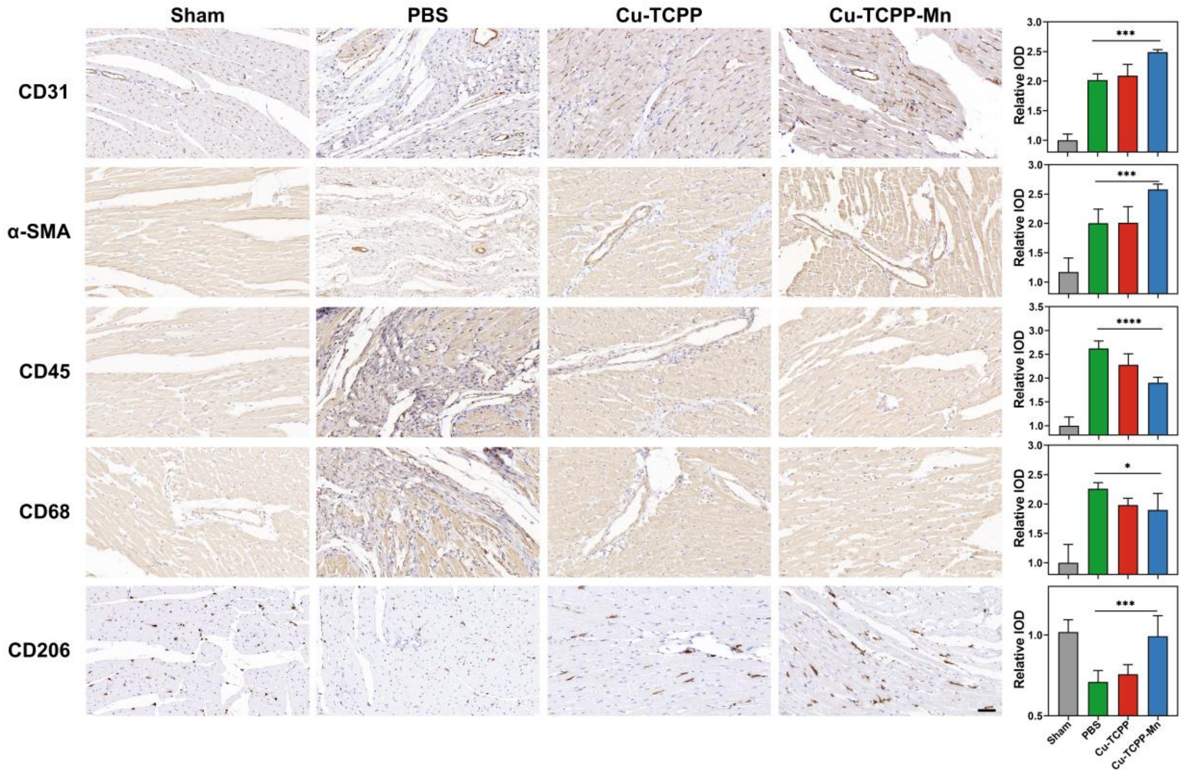

Figure S26. IHC staining and statistical analysis of heart tissues (CD31, α-SMA, CD45, CD68 and CD206) harvested from rat I/R injury models treated with Cu-TCPP and Cu-TCPP-

Mn. Scale bar, 20 μm.

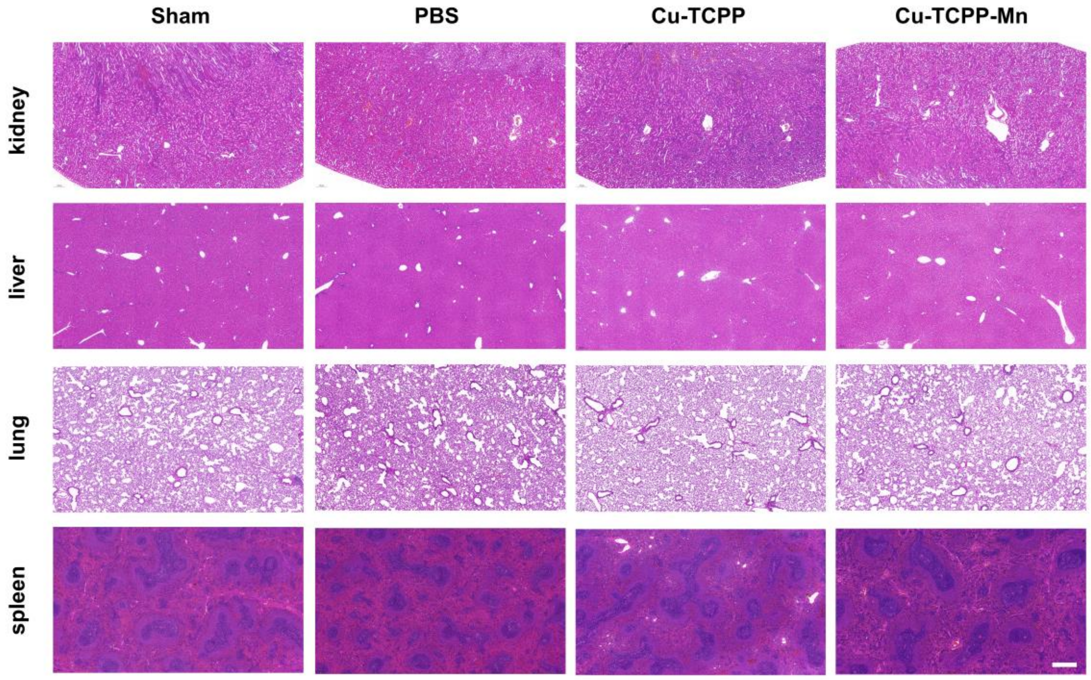

Figure S27. H&E staining of major organs tissues harvested from sham or I/R injury rat models treated with Cu-TCPP and Cu-TCPP-Mn. Scale bar, 500 μm.

Table S1. Mn and Cu content in Cu-TCPP and Cu-TCPP-Mn determined by ICP-AES.

	**Cu-TCPP-Mn**	**Cu-TCPP**
Cu (**wt.**%)	11.83%	14.13%
Mn (**wt.**%)	2.89%	

Table S2. Comparison of metal content in various single-atomic catalysts

**Materials**	**Loading of metal**	**References**
Cu-TCPP-Mn	2.89 wt.% (ICP)	This work

20Mn-NC-first	0.68 wt.% (ICP)	*Nat Catal*. 2018,1,935-945[3]
20Mn-NC-second	3.03 wt.% (ICP)	
Cu2@C3N4	0.35 wt.% (ICP)	*Nat Commun*. 2022,13:1375[4]
FeN4/GN	4.0 wt.% (ICP)	*Sci Adv*. 2015;1:e1500462[5]

Table S3. Structural parameters extracted from EXAFS fitting.

Sample	Edge	Path	CN	R(Å)	σ2(10-3 Å)	ΔE0(eV)	R-factor
Cu-TCPP-Mn	Mn-K	Mn-N	3.71	1.92	3.9	-8.1	0.014
Cu-TCPP-Mn	Cu-K	Cu-N	3.09	1.95	3.4	-8.3	0.003
		Cu-O	0.99	1.94	5.4	7.4	
Cu-TCPP	Cu-K	Cu-N	3.16	1.98	2.13	-5.9	0.009
		Cu-O	1.01	1.93	1.23	8.4	

Notes: CN, coordination number; R, distance between absorber and backscatter atoms. σ2, Debye-Waller factor; ΔE0, the inner potential difference between the reference compound and the experimental sample. R-factor, the goodness of fit.

Table S4. Comparison of CAT-like kinetics for Cu-TCPP and Cu-TCPP-Mn.

	K_m (mM)	V_{max} (mg/L/min)	Kcat (s^{-1})	K_{cat}/K_m ($mM^{-1}S^{-1}$)
Cu-TCPP	134.60	0.3135	1.044×10^6	7.756×10^3
Cu-TCPP-Mn	34.65	0.7289	2.430×10^6	7.013×10^4

References:

1. Zhao Y, Wang J, Pei R. Micron-sized ultrathin metal-organic framework sheet. J Am Chem Soc. 2020;142(23):10331-36.
2. Teng Z, Li W, Tang Y, Elzatahry A, Lu G, Zhao D. Mesoporous organosilica hollow nanoparticles: synthesis and applications. Adv Mater. 2019,31(38):e1707612.
3. Li JZ, Chen MJ, Cullen DA, Hwang S, Wang MY, Li BY, et al. Atomically dispersed manganese catalysts for oxygen reduction in proton-exchange membrane fuel cells. Nat Catal. 2018;1(12):935-45.
4. Xie P, Ding J, Yao Z, Pu T, Zhang P, Huang Z, et al. Oxo dicopper anchored on carbon nitride for selective oxidation of methane. Nat commun. 2022;13(1): 1375.
5. Deng D, Chen X, Yu L,Wu X, Liu Q, Liu Y, et al. A single iron site confined in a graphene matrix for the catalytic oxidation of benzene at room temperature. Sci Adv. 2015;1(11):e1500462.
